# Self-assembled genistein nanoparticles suppress the epithelial-mesenchymal transition in glioblastoma by targeting MMP9

**DOI:** 10.1016/j.mtbio.2025.101606

**Published:** 2025-02-27

**Authors:** Qingyu Zhao, Yong Li, Qian Sun, Ronggui Wang, Haoran Lu, Xinyi Zhang, Lun Gao, Qiang Cai, Baohui Liu, Gang Deng

**Affiliations:** a Department of Neurosurgery, Renmin Hospital of Wuhan University, Wuhan, Hubei, 430060, PR China; b Department of Neurosurgery, The Second Affiliated Hospital of Zhejiang University School of Medicine, Hangzhou, Zhejiang 310000, P.R. China

**Keywords:** Genistein, Glioblastoma, Self-assembly, Epithelial-mesenchymal transition, MMP9

## Abstract

Glioblastoma (GBM) is the most prevalent and aggressive primary malignant brain tumor in adults, known for its poor prognosis and resistance to conventional treatments. The blood-brain barrier (BBB) presents a significant challenge in delivering effective treatments. In this study, we developed a carrier-free, self-assembled nanosystem using genistein (GE), a naturally occurring isoflavone, to enhance therapeutic delivery across the BBB. GE nanoparticles (GE NPs) were synthesized via solvent emulsification evaporation, in uniform spherical particles (∼180 nm), stabilized by hydrogen bonding and π-π interactions. The GE NPs demonstrated optimal physicochemical properties, including stability, high BBB permeability, prolonged circulation time. In vitro studies revealed that GE NPs inhibited GBM cell proliferation, induced apoptosis and suppressed epithelial-mesenchymal transition (EMT) by promoting the degradation of MMP9. In vivo, GE NPs significantly reduced tumor growth and extended survival in an orthotopic GBM mouse model, outperforming temozolomide treatment. Mechanistic analysis indicated that GE NPs inhibited the degradation of the extracellular matrix by targeting the catalytic domain of MMP9, thereby effectively suppressing the EMT of GBM. This research highlights the potential of GE NPs as a novel therapeutic approach for GBM, addressing drug delivery challenges while improving anti-tumor efficacy. Further optimization for enhanced tumor retention and exploration of combination therapies may improve clinical outcomes (Graphical Abstract).

## Graphical abstract

**Graphical Abstract**. Schematic illustration of the synthesis and in vivo biological processes of genistein nanoparticles.

**Figure:**
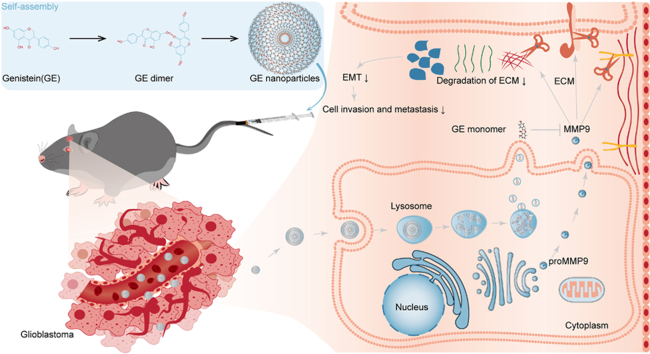

## 1 Introduction

Glioblastoma (GBM), the most common and malignant primary malignant brain tumors in adults according to the 2021 World Heathy Organization (WHO) [1]. It is accounting for 50.1 % among primary malignant central nervous system tumors, and 14.2 % among all primary brain tumors [2]. Across generations, a multimodal treatment regimen is employed in the fight against GBM, which combines maximal safe surgical resection with adjuvant radiotherapy and chemotherapy. Nonetheless, GBM patients have the lowest median survival of 8 months, with only 6.9 % surviving five years post-diagnosis [2]. Hindered by the blood-brain barrier (BBB) of the central nervous system, the primary challenge in the development of novel drugs for GBM lies in effectively delivering therapeutic agents to specific target sites [3]. Temozolomide (TMZ), as a lipophilic agent, can readily penetrate the BBB to induce cell death through DNA damage, making it the first-lines chemotherapy in clinical management of GBM [4]. Nonetheless, most patients demonstrate decreased responsiveness to TMZ, which may be linked to DNA repair mechanisms such as O-6-methylguanine-DNA methyltransferase (MGMT) [5], metabolic reprogramming [6], and the presence of GBM stem-like cells (GSCs) [7]. Consequently, there is a pressing need to identify new anticancer agents capable of effectively crossing the BBB and reaching tumor tissues to enhance therapeutic outcomes.

Genistein (GE), a naturally occurring isoflavone predominantly found in soy-based products, has emerged as a compound with significant therapeutic applications, particularly in the field of oncology [8]. Acting as a phytoestrogen, GE demonstrates diverse biological functions such as antioxidant, anti-inflammatory, and anticancer activities [9]. GE demonstrates broad-spectrum antitumor efficacy through mechanisms including proliferation inhibition, apoptosis induction, and cell cycle arrest in breast, colorectal, and prostate cancers, with particular promise in suppressing tumor cell invasion and migration [[10], [11], [12], [13], [14]]. In GBM models, preclinical studies have confirmed its radio sensitizing effects when combined with low-dose radiation and its capacity to counteract radiation-induced metastatic activation. However, the clinical application of GE is severely limited by its suboptimal bioavailability (<10 %). Nanoencapsulation strategies have been explored to overcome this pharmacokinetic barrier, including co-delivery with TMZ in poly(lactic-co-glycolic acid) (PLGA) nanoparticles showing enhanced in vitro anti-GBM activity (albeit without in vivo validation) [15]. Current nano-delivery systems face dual challenges: potential toxicity from carrier degradation byproducts and compromised systemic retention due to rapid renal clearance, underscoring the need for advanced engineering solutions to optimize therapeutic payload delivery.

Supramolecular self-assembly is the spontaneous organization of a defined number of small molecular monomers into entities with specific microstructures, including spheres, vesicles, fibrillary networks, multilayered sheet networks, and flower-like networks, ranging in size from nano-to micro-meter. In comparison to complex drug modifications, the process of small molecule self-assembly is relatively simple and direct, requiring no addition of substances metabolically challenging for the body. Consequently, various supramolecular structures formed through the self-assembly of natural small molecules have been widely applied in fields such as drug delivery, capturing pollutants, and the synthesis of nanomaterials [[16], [17], [18], [19]]. Using the solvent emulsification evaporation method, we have assembled certain known natural drug small molecules into entities with specific structures, facilitating their enhanced penetration of the BBB for targeted therapeutic effects [[20], [21], [22]].

To address the aforementioned issues, we developed a carrier-free self-assembled nano-system for GBM treatment. It exhibited excellent BBB permeability and cellular internalization efficiency. Additionally, they demonstrated significant effects in suppressing EMT, cell proliferation and promote apoptosis in GBM cells. We further confirmed that the catalytic domain of MMP9 is a critical target of GE NPs in the degradation of the extracellular matrix, thereby inhibiting the EMT in GBM. In conclusion, this study successfully synthesized a carrier-free self-assembled GE nano-system, elucidated the underlying mechanisms of GE's therapeutic effects against GBM, and provided novel insights into GBM treatment strategies.

## 2 Materials and methods

### 2.1 Antibody and reagents

GE was purchased from Shanghai Aladdin Biochemical Technology Co., Ltd. (Shanghai, China). The antibodies used for immunostaining were anti-rabbit-cleaved-Caspase3 (25128-1-AP, Proteintech); anti-rabbit-Caspase3/p17/p19 (19677-1-AP, Proteintech); anti-mouse-Bcl-2 (68103-1-Ig, Proteintech), anti-rabbit-N-cadherin antibody (22018-1-AP; Proteintech); anti-rabbit-Vimentin antibody (5741; CST); anti-rabbit-MMP9 antibody (10375-2-AP, Proteintech); anti-rabbit-Snail antibody (14-9859-82, Invitrogen); anti-rabbit-beta Catenin (S1067-2-AP, Proteintech) antibody; anti-rabbit-AKT antibody (342529, Zenbio); anti-rabbit-Phospho-AKT (ser473) antibody (381555, Zenbio); anti-rabbit-mTOR antibody (28273-1-AP, Proteintech); anti-rabbit- Phospho-mTOR antibody (80596-1-RR, Proteintech); anti-rabbit-beta actin antibody (GB15003-100; Servicebio).

### 2.2 Synthesis of GE NPs

GE NPs were prepared using a standard emulsion process. First, 10 mg of GE was dissolved in 1 ml of a mixture of ethyl acetate, methanol, and ethanol in an 8:1:1 ratio. This solution was then added dropwise, while vortexing, to a 50 ml tube containing 3 ml of Pluronic F-127 (P2443, Sigma-Aldrich, molecular weight = ∼12600 g/mol) solution (2.5 wt%). Next, the emulsion was sonicated on ice for a total of 150 s (power:120W, 10s on, 10s off). The resulting mixture was poured into a beaker containing 30 ml of Pluronic F-127 solution (0.3 wt%) under vigorous stirring. The mixture was stirred overnight at room temperature. Following this, the nanoparticles were collected by centrifugation at 18,000 rpm for 30 min at 4 °C. The nanoparticles were washed three times with 30 ml of water, each time collected by centrifugation (18,000 rpm at 4 °C for 30 min). Finally, the nanoparticles were resuspended in 1 ml of distilled water and stored. GE-C6 NPs, encapsulating Coumarin 6, and GE-DiD NPs, encapsulating the Far-red Plasma Membrane Fluorescent Probe DiD, were prepared using the same method.

### 2.3 Dynamic light scattering (DLS)

The GE nanoparticles (GE NPs) were diluted to an aqueous solution with a concentration of 1 mg/ml. The hydrodynamic diameter and zeta potential were then measured using a dynamic light scattering (DLS) instrument (Zetasizer Nano ZSP, Malvern Instruments Ltd., UK).

### 2.4 Transmission electron microscope (TEM)

GE NP suspensions were deposited onto ultrathin carbon films (Zhongjingkeyi, China) and allowed to adsorb for several minutes. The grids were subsequently placed in a fume hood and left to air-dry completely. After drying, the grids were transferred to a transmission electron microscope (JEM-F200, JEOL, Japan) for high-resolution imaging to analyze the nanoparticles' morphology, particle size, and spatial distribution.

### 2.5 Scanning electron microscopy (SEM)

Gold coating of the samples was conducted using a sputter coater (auto fine coater JFC-1200, JEOL, Japan) under vacuum and an argon atmosphere, with a sputtering current of 40 mA applied for 60 s. Scanning electron microscopy (SEM) analysis was performed using a Tescan MIRA SEM system (Tescan, Czech Republic) at an acceleration voltage of 5 kV and a magnification of 50,000 × .

### 2.6 ^1^H nuclear magnetic resonance (^1^H NMR)

The ^1^H NMR spectra of GE and GE NPs were recorded on a Bruker AVANCE™ NEO 600 MHz spectrometer (Bruker, Switzerland), using DMSO-d6 as the solvent and tetramethyl silane (TMS) as the internal standard reference.

### 2.7 X-ray diffraction (XRD)

XRD analysis of bulk GE and GE NPs was conducted using a XtaLAB Synergy Custom Single Crystal X-ray Diffractometer (Rigaku, Japan). Data acquisition was performed over a 2θ range of 8°–80°.

### 2.8 Fourier transform infrared (FTIR) spectra

FTIR spectra of bulk GE and GE NPs were obtained using a NICOLET 5700 FT-IR Spectrometer (Thermo Fisher Scientific, USA). Measurements were taken across a wavenumber range of 4000 cm^−1^ to 400 cm^−1^with a spectral resolution of 4 cm^−1^.

### 2.9 High-performance liquid chromatography (HPLC)

The stability profile of GE NPs was systematically investigated under physiological conditions (PBS, pH 7.4, 37 °C) using an in vitro dialysis model. Firstly, 2 mL of NP suspension (7.5 mg/mL) was loaded into a dialysis membrane (MWCO 3.5 kDa) and immersed in 20 mL PBS maintained at 37 °C with continuous agitation (150 rpm) using a magnetic stirring system. Aliquots (500 μL) were periodically withdrawn at predetermined intervals (3, 6, 12, 24, 48, 72, and 96 h) with simultaneous replenishment of equal volume fresh PBS. The collected samples were lyophilized and reconstituted in 50 μL 90 % ethanol prior to chromatographic analysis. Quantification of GE content was performed using an Agilent 1260 Infinity II HPLC system (America) equipped with a Sion Chrom ODS-BP column (4.6 × 250 mm, 5 μm). Chromatographic separation was achieved with a binary mobile phase consisting of: Mobile phase A: 0.1 % (v/v) acetic acid in ultrapure water, Mobile phase B: 0.1 % (v/v) acetic acid in acetonitrile. The injection volume was set at 2 μL with UV detection wavelength fixed at 260 nm. All experiments were conducted in triplicate to ensure statistical reliability.

### 2.10 Density functional theory (DFT) simulations

DFT calculations were performed using Gaussian 16 suite of programs, and results were analyzed with the Multiwfn_3.7 software (win64 version). All molecular structures were fully optimized at the B3LYP/6-31g (d) level of theory. Calculation of interaction energies. The interaction energy (ΔE) is expressed by the energy difference between the complex (E_GE&GE_) and the monomers (E_GE_). Firstly, optimizing the monomers GE geometric structure and frequency calculation at B3LYP/6-31g (d) level to obtain the thermodynamic energy correction value and zero-point energy correction (ZPEC). Secondly, optimizing the GE&GE geometric structure and frequency calculation at B3LYP/6-31g (d) level. Therefore, the different interaction energies are calculated:

ΔE = E_GE&GE_ - E_GE_

where E_GE&GE_, E_GE_ are the complex (GE&GE) energy, monomer GE (total electronic energy), respectively.

### 2.11 Collection of GBM-related gene targets

Gene expression data comprising 168 GBM tissues and 5 normal tissues from The Cancer Genome Atlas (TCGA), along with 1152 normal brain tissues from the Genotype-Tissue Expression (GTEx) database, were obtained from the University of California, Santa Cruz Xena (UCSC Xena) platform (https://xena.ucsc.edu/). Differentially expressed genes in GBM were identified using the R package limma (version 3.56.2), employing screening criteria of a log2Fold Change >2 and a p-value (Padj) < 0.05.

### 2.12 Prediction of drug targets for GE

The SMILES format of GE was retrieved from PubChem (https://pubchem.ncbi.nlm.nih.gov/) and subsequently submitted to the SwissTargetPrediction (STP, http://www.swisstargetprediction.ch/), SuperPRED (SP, https://prediction.charite.de/), and PharmMapper (PM, http://lilab-ecust.cn/pharmmapper/) databases. These platforms were used to predict the potential targets of GE for further analysis.

### 2.13 PPI network construction and functional enrichment analysis of GBM-GE intersecting genes

The protein-protein interaction (PPI) network for the intersecting genes between GBM and GE was generated using the STRING database (https://string-db.org/), with a minimum interaction score set above 0.4. The PPI network was analyzed, key hub genes were identified, and visualized using Cytoscape 3.9.0 software. Gene Ontology (GO) and Kyoto Encyclopedia of Genes and Genomes (KEGG) enrichment analyses were conducted using the ClusterProfiler package in R (version 3.18).

### 2.14 Molecular docking analysis

Molecular docking simulations of GE and the target protein were performed using AutoDock software. First, the target protein structure was downloaded from the PDB database and prepared using AutoDock Tools (ADT) by removing water molecules and ligands. The structure of GE was obtained from PubChem and optimized. Both the protein and GE structures were converted to PDBQT format, and a grid box was set to define the region of the protein's active site. Docking calculations were carried out using AutoDock Vina, generating binding modes and binding energies. Finally, the docking results were analyzed using PyMOL, and the binding mode with the lowest binding energy was selected and saved for further use.

### 2.15 Molecular dynamics (MD)

Molecular dynamics (MD) simulations of the GE-MMP9 complex were performed using GROMACS 2023 for a total of 100 ns. The CHARMM 36 force field was applied to the protein [23], while the topology for the ligand was generated using the GAFF2 force field. Periodic boundary conditions were employed, and the GE-MMP9 complex was placed in a cubic simulation box. The box was filled with water molecules using the TIP3P water model [24]. Electrostatic interactions were handled using the Particle Mesh Ewald (PME) method, while the Verlet algorithm was used for force calculations. The system underwent 100,000 steps of equilibration under both NVT (constant number of particles, volume, and temperature) and NPT (constant number of particles, pressure, and temperature) ensembles. The coupling constant was set to 0.1 ps with a total equilibration time of 100 ps. Both van der Waals and Coulomb interactions were calculated with a 1.0 nm cutoff. Finally, the production MD simulation was carried out for 5,000,000 steps with a 2 fs timestep, corresponding to a total simulation time of 100 ns under constant temperature (300 K) and constant pressure (1 bar) using GROMACS 2023.

### 2.16 Cell culture and animal studies

The U87MG human GBM cell line, along with the GL261 mouse glioma and bEnd.3 mouse brain endothelial cell lines, were cultured at 37 °C in a 5 % CO2 humidified incubator. Cells were grown in high-glucose DMEM (Gibco, USA) supplemented with 10 % fetal bovine serum (FBS, Gibco, USA), 100 U/mL penicillin, and 100 μg/mL streptomycin. C57B/6J mice of 6–8 weeks, and female SD rats of 180–200 g, were purchased from Guangdong Medical Laboratory Animal Center. And the mice were housed under normal specific pathogen-free (SPF) conditions.

### 2.17 Cell viability assay

The anti-proliferative activity of GE and GE NPs against U87 and GL261 cells was assessed using the Cell Counting Kit-8 (CCK8) assay (Beyotime Institute of Biotechnology, China). U87 cells were initially seeded in 96-well plates at a density of 10,000 cells per well and allowed to adhere for 24 h. After this period, the medium was replaced with fresh DMEM containing either GE or GE nanoparticles at concentrations of 1, 2, 4, 8, 16, 32, 64, and 128 μg/ml. After 24 h of treatment, 10 μl of CCK8 solution was added to each well, followed by incubation at 37 °C for 1 h. Absorbance at 450 nm was then measured using a microplate reader to evaluate cell viability.

### 2.18 Edu DNA synthesis analysis

Cell growth was evaluated using the BeyoClick™ EdU-488 Kit (Beyotime Institute of Biotechnology, China). U87 cells were seeded into 96-well plates at a density of 10,000 cells per well in 100 μL of DMEM. The cells were treated with GE NPs at concentrations of 0, 5, 10, and 20 μg/mL for 24 h. After the treatment, 50 μL of EdU medium was added to each well, followed by a 2-h incubation. The cells were then fixed with 4 % paraformaldehyde for 30 min. Following fixation, 100 μL of the 1x Click reaction mixture was added and incubated for 30 min. Finally, the cells were counterstained with DAPI in the dark for 30 min. Fluorescence images of DAPI and EdU staining were captured using a fluorescence microscope (Olympus DP74) to assess cell growth.

### 2.19 TUNEL staining

The Roche In Situ Cell Death Detection Kit (TMR Red) was used to detect apoptosis in U87MG cells grown on coverslips in a 6-well plate. 1 × 10^5 U87MG cells were seeded onto sterile coverslips placed at the bottom of each well and incubated until they reached 50–60 % confluence. Cells were then treated with GE NPs (0, 5, 10, 20 μg/ml) for 24 h. After treatment, the cells were fixed with 4 % paraformaldehyde (PFA) for 30 min and permeabilized using 0.2 % Triton X-100 in PBS for 10 min. Following permeabilization, 50 μl of TUNEL reaction mixture was added to each coverslip and incubated in a humidified chamber at 37 °C for 30 min in the dark. After incubation, cells were stained with 1 × DAPI for 30 min to visualize nuclei. Coverslips were then mounted onto glass slides, and fluorescence images were captured using a microscope (Olympus DP74). DAPI-stained nuclei were visualized in blue, while TUNEL-positive cells showed red fluorescence, with an excitation wavelength of 520–560 nm and an emission wavelength of 570–620 nm.

### 2.20 Mitochondrial membrane potential (mtΔΨ) analysis

For mtΔΨ analysis, the JC-1 Mitochondrial Membrane Potential Assay Kit (Beyotime, China) was used according to the manufacturer's protocol. First, U87 cells were seeded in 6-well plates at a density of 1 × 10^5^ cells per well and allowed to adhere overnight. After adherence, the cells were treated with GE NPs at concentrations of 0, 5, 10, and 20 μg/mL for 24 h. Following treatment, the cells were washed with PBS and then incubated with JC-1 solution at 37 °C for 20 min in the dark. In cells with high mtΔΨ, red fluorescence (J-aggregates) was emitted, whereas in cells with low mtΔΨ, green fluorescence (JC-1 monomers) was observed. Subsequently, the cells were washed with JC-1 buffer and immediately examined under a fluorescence microscope (Olympus DP74). Finally, the mtΔΨ was calculated as the ratio of red to green fluorescence intensity by image J.

### 2.21 Cell migration and invasion

To evaluate cell migration and invasion, U87 cells were exposed to varying concentrations of GE NPs (0, 5, 10, and 20 μg/ml) for 24 h prior to the assays. Migration was assessed using a wound healing assay. U87 cells were cultured in 6-well plates until they reached 90 % confluence. A straight scratch was introduced into the cell monolayer using a pipette tip, followed by PBS washes to remove cellular debris. Fresh serum-free medium containing the specified concentrations of GE NPs was then added. Images of the wound area were taken at 0, 24 and 48 h using a light microscope to monitor cell migration into the scratch. For the invasion assay, Transwell chambers with 8 μm pores were utilized. The upper chamber was coated with Matrigel, and U87 cells (2 × 10^4^ per well) were seeded in serum-free medium containing GE NPs at 0, 5, 10, and 20 μg/ml. The lower chamber was filled with complete medium containing 10 % FBS, serving as a chemoattractant. After 24 h of incubation at 37 °C, non-invading cells on the upper surface of the membrane were gently removed with a cotton swab. Invading cells on the lower surface were fixed with 4 % paraformaldehyde, stained with crystal violet, and counted under a light microscope.

### 2.22 Western blot analysis

U87MG cells were lysed on ice for 30 min using modified RIPA buffer (No. p0013b, Beyotime Biotechnology, China) to facilitate thorough protein extraction. The lysates were centrifuged at 12,000 rpm for 15 min at 4 °C to remove cellular debris. Protein concentrations were measured using the BCA Protein Assay Kit (Beyotime Biotechnology, China) following the manufacturer's instructions. After quantification, the lysates were denatured by heating at 100 °C for 5 min and subsequently mixed with loading buffer. Equal protein amounts, verified by the BCA assay, were loaded onto 8–12 % SDS-PAGE gels for electrophoresis, enabling protein separation based on molecular weight. Post-electrophoresis, proteins were transferred onto a nitrocellulose membrane using a wet transfer system at 4 °C. The membrane was then blocked with 5 % skimmed milk in TBS-T (Tris-buffered saline with 0.1 % Tween-20) for 1 h at room temperature to prevent nonspecific binding. After blocking, the membrane was incubated with primary antibodies overnight at 4 °C, followed by a 1-h incubation with secondary antibodies at room temperature.

### 2.23 Immunofluorescence stained

Cells were fixed in 4 % paraformaldehyde in PBS for 30 min at room temperature, followed by permeabilization with 0.2 % Triton X-100 in PBS for 15 min. After three washes with PBS (5 min each), the slides were blocked with 1 % BSA in PBS for 30 min to minimize nonspecific binding. The slides were then incubated overnight at 4 °C with a diluted primary antibody in a humidified chamber. After PBS washing, a secondary antibody (Antkin, Wuhan, China) was added and incubated for 1 h at 37 °C in the dark to preserve fluorescence. Subsequently, the nuclei were counterstained with DAPI, and the slides were mounted using an antifade medium. Fluorescence images were acquired with a fluorescence microscope (Olympus BX51, Japan).

### 2.24 Protein stability experiment and degradation assay

The stability of MMP9 protein in U87 and GL261 cells was assessed using Cycloheximide (CHX), a protein synthesis inhibitor (66-81-9, TargetMol, 40 μg/mL). Cells were treated with CHX for varying durations ranging from 0 to 1 h. Following this, the cells were treated with the proteasome inhibitor MG132 (133407-82-6, MedChemExpress, 40 μM) for 1 h. A negative control group consisted of cells treated with dimethyl sulfoxide (DMSO).

### 2.25 In vitro BBB model

An in vitro BBB model was developed using bEnd.3 cells cultured on Transwell polyester membranes in 24-well plates. Briefly, bEnd.3 cells (2 × 10^4^ cells per well) were seeded in the upper chamber of the Transwell inserts and allowed to grow for 4 days until confluence. Transendothelial electric resistance (TEER) was monitored daily using the Millicell ERS-2 (Millipore, USA). Once the TEER exceeded 200 Ω cm^2^, indicating an intact barrier, GL261 cells (2 × 10^4^ cells per well) were seeded on glass coverslips placed in the lower chamber and co-cultured with the bEnd.3 monolayer overnight. Coumarin-6-labeled GE nanoparticles (GE-C6 NPs) were prepared, and 200 μl of fresh medium containing either free dye or GE-C6 NPs (10 μg/mL) was added to the upper chamber. After 12 h of incubation, both bEnd.3 and GL261 cells were washed twice with PBS, fixed with 4 % paraformaldehyde for 15 min at room temperature, and stained with DAPI for 10 min to visualize the nuclei. The uptake and translocation of GE NPs were then assessed using laser scanning confocal microscopy (LSCM).

### 2.26 Intracellular localization of GE NPs

GL261 cells were seeded at a density of 3 × 10^5^ cells per well in confocal dishes and incubated at 37 °C in a 5 % CO_2_ humidified incubator for 24 h to allow cell adhesion. After 24 h, GE NPs (labeled with C6 or DiD) were introduced into the wells and incubated for 1 h at 37 °C. Following the incubation, the cells were washed three times with PBS (5 min per wash) to remove any uninternalized nanoparticles. Subsequently, pre-warmed Lyso-Tracker Green working solution (Servicebio, G1722) was added according to the manufacturer's instructions, and the cells were incubated at 37 °C for an additional 40 min to stain the lysosomes. After staining, the solution was carefully removed, and the cells were thoroughly washed three times with PBS. The intracellular localization of GE NPs and lysosomes was observed using LSCM.

### 2.27 Construction of GL261-luc cell line

To develop the GL261-luc cell line, we began by preparing lentiviral vectors that encode luciferase. These vectors were then used to transfect 293T cells, along with packaging plasmids, to produce lentivirus. After 48 h, the lentivirus was harvested and concentrated. Next, GL261 cells were plated in a 6-well plate and transduced with the concentrated viral solution, which included polybrene, for 24–48 h. Post-transduction, we selected for stable integration of the virus using puromycin and expanded the resulting cells. To confirm successful transduction, we performed a luciferase assay and, if necessary, fluorescence microscopy. Finally, the GL261-luc cells were cryopreserved for future use.

### 2.28 Intracranial xenograft model

All animal experiments were performed in accordance with the guidelines of the Animal Experiment Center at the First Clinical College of Wuhan University (Wuhan, China). Male C57BL/6J mice, aged 6–8 weeks, were housed in a sterile environment for the study, which was approved by the Animal Protection and Utilization Committee of the People's Hospital of Wuhan University. GL261-luc cells, harvested during their logarithmic growth phase, were resuspended in PBS to a concentration of 1 × 10^5 cells/μl. Following anesthesia with isoflurane, a 1–2 mm craniotomy was performed over the right frontal lobe of the mouse. Using stereotaxic techniques, 1 × 10^5 GL261 cells were injected into the right ventricle of each mouse. Post-surgery, the mice were given appropriate care, including antibiotic treatment and close monitoring for any signs of distress. Tumor progression was monitored through regular observations and luciferase bioluminescence imaging system detection.

### 2.29 In vivo pharmacokinetics analysis

Tumor-free Sprague-Dawley (SD) rats (approximately 200 g) were randomly divided into two groups (n = 3 per group) and received either free C6 or GE-C6 NPs via slow intravenous injection. Blood samples were collected at predetermined time points: 1, 3, 6, 9, 12, 24, 48 h post-injection. The samples were centrifuged to separate the plasma. C6 was extracted from the plasma using methanol, and the resulting supernatant was analyzed using a microplate reader with excitation at 450 nm and emission at 500 nm to quantify the concentration of C6. The concentration of C6 in the GE-C6 group at 0.25 h post-injection was used as a reference (set to 100 %), and the relative concentrations of C6 in all other groups were calculated accordingly. GL261-Luc glioma-bearing C57BL/6J mice with similar tumor sizes were randomly divided into four groups (n = 3 per group). GE-C6 NPs were administered via tail vein injection at 1, 6, 24, and 48 h before in vivo imaging. At the designated time points, the mice were euthanized and perfused with saline to remove circulating blood. Major organs were collected, and the fluorescence intensity of C6 was measured using an IVIS imaging system to assess its biodistribution over time.

### 2.30 In vivo biodistribution and targeting ability

To evaluate the ability of GE NPs to bypass the BBB and penetrate GBM, GL261-Luc glioma-bearing C57BL/6J mice were intravenously injected via the tail vein with free C6, GE-C6 NPs, and PLGA-C6 NPs. At 8 h post-injection, the mice were euthanized, and the brain, including the tumor, as well as key organs (heart, liver, spleen, lungs, and kidneys), were harvested. Fluorescence intensity of C6 in these tissues was measured using an IVIS imaging system to assess the biodistribution and relative accumulation of the nanoparticles.

### 2.31 Determining the efficacy of GBM treatment

On the seventh day after cell implantation, mice were randomly assigned to four groups: PBS, GE, TMZ, and GE NPs. Each group received tail vein injections of the respective treatments every two days for a total of six injections. Tumor growth was monitored every five days using the IVIS system, with simultaneous recording of neurological symptoms and body weight. Mice were euthanized if body weight decreased by more than 20 %. After euthanasia, mice were perfused with 4 % paraformaldehyde, and key organs were harvested for H&E staining. The brains were then excised, weighed, and fixed in 4 % paraformaldehyde for paraffin embedding, followed by immunofluorescence and immunohistochemistry analysis.

### 2.32 Statistical analysis

All results are presented as the mean ± standard deviation (SD), with each experiment conducted in triplicate. For statistical evaluation, one-way ANOVA followed by Bonferroni correction was employed for single-factor comparisons, whereas two-way ANOVA with Bonferroni correction was applied for multi-factorial analyses. Statistical significance was denoted as P < 0.05 (∗), P < 0.01(∗∗), and P < 0.001(∗∗∗). All statistical charts were created using GraphPad Prism 8.0. A p value of less than 0.05 was considered as statistical significance.

## 3 Result and discussion

### 3.1 Synthesis and Characterization of GE NPs

GE (5,7,4′-trihydroxyisoflavone) is a multifunctional natural isoflavone compound with a 15-carbon skeleton. Similar to other plant components with estrogenic activity (such as lignans), GE is a typical phytoestrogen. It was first isolated from GE tinctoria in 1899, from which it derives its name. Owing to its structural similarity to estradiol, GE has an affinity for binding to estrogen receptors. It exhibits high solubility in polar solvents such as DMSO, acetone, and ethanol, but low solubility in water [25]. Compared to its metabolites, free GE shows lower bioavailability and lower concentrations in plasma and tissues in both animal models and human studies. After consuming a soy-based diet, over 90 % of GE in human urine samples is present in glucuronide or sulfate forms, with only 0.9 % existing in its free form. The poor oral bioavailability of GE is mainly attributed to its low water solubility, extensive metabolism in the gastrointestinal tract, and efflux by transport proteins such as breast cancer resistance protein (BCRP) [26,27].

**Fig. 1:**
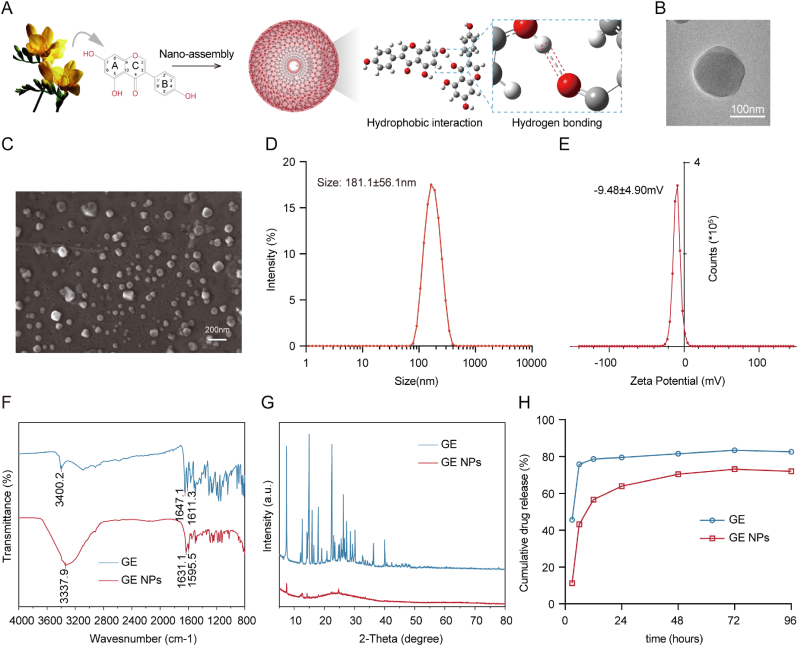
**Synthesis and Characterization of GE NPs**. (A)Scheme of self-assembly process and DFT simulations from GE to GE NPs; (B) Representative TEM image of GE NPs; (C) Representative SEM image of GE NPs; (C, D) Mean sizes and surface charge of GE NPs detected by DLS; (F) FTIR of free GE and GE NPs; (G) XRD of free GE and GE NPs; (H) Drug release of GE NPs. All results are presented as mean ± standard deviation (SD). n = 3 per group.

Numerous studies have explored methods to improve GE delivery, confirming the potential of nanotechnology in cancer treatment and making it a significant area of research. In previous studies, our team successfully prepared various natural small-molecule nanoparticles with strong BBB penetration and tumor-targeting abilities using the solvent emulsification-evaporation method. Building on this approach, we successfully self-assembled free GE monomers into GE NPs and analyzed the process using DFT simulations. The DFT calculations revealed an interaction energy between the GE molecules of ΔE = −14.19 kcal/mol, indicating a decrease in system energy. This negative energy change suggests that the self-assembly is exothermic, likely promoting molecular binding or interactions. Furthermore, a hydrogen bond forms between the carbonyl group (C=O) at the 4th position of one molecule and the hydroxyl group (O-H) at the 7th position of the other, further stabilizing the complex (Fig. 1A). Through ^1^H NMR spectrum, we found that GE NPs is mainly composed of GE component (Fig. S1, Supporting Information). TEM and SEM revealed that these nanoparticles are uniform spherical structures with a size of approximately 150 nm (Fig. 1B–C). Generally, spherical particles in the 80–200 nm range are considered to have good stability and safety, indicating that the size of GE NPs is ideal. Further DLS analysis showed that the average hydrodynamic diameter of GE NPs was 181.1 ± 56.1 nm (Fig. 1D). The average surface charge was −9.48 ± 4.90 mV, consistent with the surface charge of red blood cells, suggesting that GE NPs are less likely to be adsorbed by red blood cells in circulation, thus extending their blood circulation time (Fig. 1E).

To explore the mechanism of GE NPs formation, FTIR was used to compare the infrared absorption peaks of free GE and GE NPs. Free GE exhibited characteristic peaks at 3400 cm^−1^, 1647 cm^−1^, and 1611 cm^−1^, corresponding to the stretching vibrations of O-H, C=O, and conjugated C=C bonds, respectively. In contrast, GE NPs displayed shifted peaks at 3333.7 cm^−1^, 1631.1 cm^−1^, and 1595.5 cm^−1^, indicating a red shift (Fig. 1F). This suggests that more intermolecular hydrogen bonds and π-π conjugation were formed within GE NPs compared to free GE. Together with DFT calculations, we infer that hydrogen bonding and π-π stacking are crucial for the self-assembly of GE into GE NPs. To further clarify the properties of GE NPs, XRD was performed to assess the crystalline structures of free GE and GE NPs within the 5–80° 2θ range. Free GE exhibited sharp characteristic peaks at 2θ = 7.4°, 14.9°, and 22.5°, indicating a higher degree of crystallinity and a more orderly molecular arrangement (Fig. 1G). In contrast, these peaks were largely absent in GE NPs, suggesting a more disordered molecular structure in the self-assembled nanoparticles. Furthermore, we evaluated the stability of GE NPs by measuring particle size after incubation in PBS for 48 and 96 h. The size remained stable at approximately 180 nm, confirming the excellent stability of GE NPs in PBS (Fig. S2, Supporting Information). Similarly, they remained maintained excellent stability in 30 % FBSsolution (Fig. S3, Supporting, Information). In vitro drug release showed that GE rapidly reached equilibrium within 3 h, whereas under the same condition, only 63 % of GE was released from GE NPs at 24 h (Fig. 1H). This suggested that GE NPs maintain stability while slowly releasing under PBS conditions.The successful self-assembly of GE into GE NPs, characterized by optimal size, stability, and a disordered molecular structure, suggests enhanced bioavailability and prolonged circulation time, addressing key challenges of free GE.

### 3.2 The cellular uptake, BBB penetration, pharmacokinetics and tumor targeting of GE NPs

We further investigated the cellular uptake, intracellular distribution, in vitro BBB penetration, in vivo pharmacokinetics, and tumor-targeting ability of GE NPs. To accurately track the location of GE NPs both in vitro and in vivo, we successfully encapsulated Coumarin-6 (C6) to create GE-C6 NPs. TEM confirmed that these nanoparticles had a similar size to the original GE NPs, measuring approximately 210 nm (Fig. S4, Supporting Information).

**Fig. 2:**
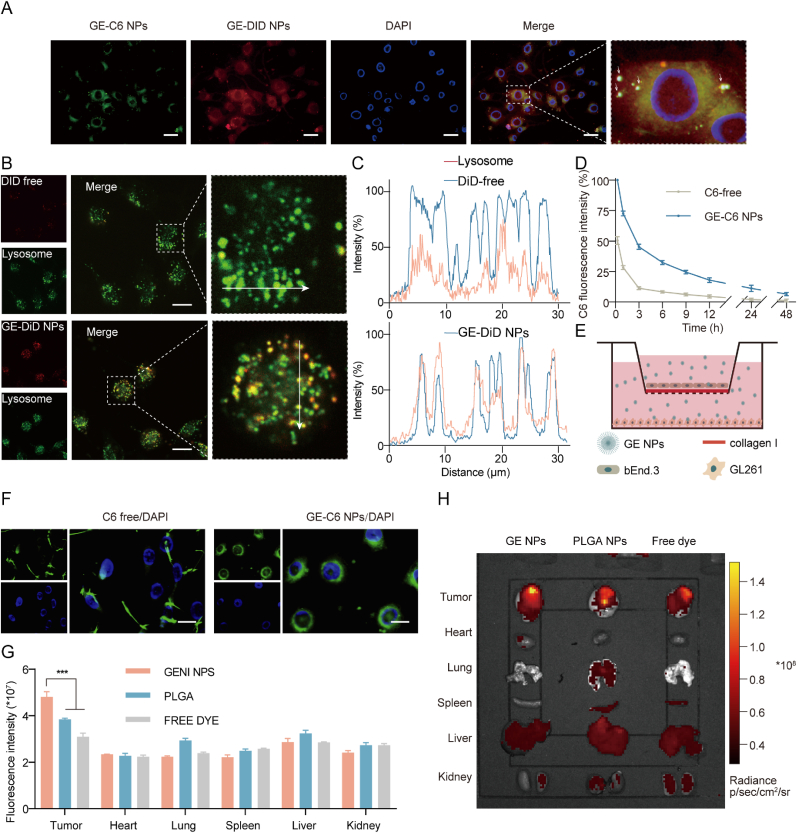
**The cellular uptake, BBB penetration, pharmacokinetics and tumor targeting of GE NPs**. (A) Fluorescence image of GE-C6 NPs and GE-DiD NPs co-localization, Scale bar = 40 μm; (B,C) Fluorescence image and intensity of DiD free, GE-DiD NPs or lysosome co-localization; Scale bar = 20 μm (D) Fluorescence levels of C6 free and GE-C6 NPs in the bloodstream over time; (E) Schematic illustration of in vitro BBB model; (F) Fluorescence image of the permeability of C6 free and GE-C6 NPs; Scale bar = 20 μm; (G, H) Ex vivo fluorescence imaging of the harvested organs by an IVIS system. Data are presented as mean ± SD. n = 3 per group, (∗∗∗p < 0.001).

Given the high expression of integrin αvβ3, we used mouse GBM cells (GL261) for our experiments and monitored cellular uptake with fluorescence microscopy. GE NPs were mainly localized in the cytoplasm, showing a more uniform distribution and stronger fluorescence signal compared to the scattered pattern of free C6 crystals (Fig. S5, Supporting Information). Quantitative analysis showed that by 40 min, about 45 % of cells had internalized GE NPs, increasing to nearly 90 % by 60 min (Fig. S6, Supporting Information). To further map the intracellular localization, we labeled GE NPs with DiD (GE-DiD NPs) and observed that they co-localized with GE-C6 NPs in the same regions within the cells (Fig. 2A). Mitochondrial and lysosomal tracers were used to identify these organelles, with mitochondria emitting red fluorescence and lysosome emitting green fluorescence. While there was no significant overlap between GE-C6 NPs and mitochondria (Fig. S7, Supporting Information), GE-DiD NPs did co-localize with lysosomes, suggesting that they primarily accumulated there and likely degraded into GE monomers (Fig. 2B–C). To assess BBB penetration, we set up an in vitro model using bEnd.3 cells cultured on collagen I-coated transwell inserts in the upper chamber and GL261 cells cultured on coverslips in the lower chamber (Fig. 2E). GE-C6 NPs showed significantly higher translocation across the endothelial layer compared to free C6 (Fig. 2F). Quantitative fluorescence analysis in GL261 cells indicated that GE-C6 NPs achieved three times the intensity of free C6 after crossing the BBB, suggesting that they not only penetrated the BBB effectively but also remained available for cellular uptake (Fig. S8, Supporting Information).

In vivo, we administered intravenous injections of either free C6 or GE-C6 NPs to SD rats and measured C6 levels in the bloodstream at different time points. While 90 % of free C6 was eliminated within 3 h, it took 24 h for GE-C6 NPs to reach a similar level of clearance (Fig. 2D). This indicates that GE-C6 NPs have a markedly longer circulation time and sustain higher plasma concentrations over the same period compared to free C6. To evaluate tumor-targeting efficiency, we used PLGA-C6 as a control and compared tumor accumulation in mice 6 h after intravenous injection of GE-C6 NPs, PLGA-C6, or free C6. Major organs were collected and imaged using IVIS (Fig. 2H). The results showed that GE-C6 NPs predominantly accumulated in the liver and tumors, with significantly greater concentration in the tumor center compared to PLGA-C6 and free C6 (Fig. 2G). These findings indicate that GE NPs possess excellent penetration capabilities across the BBB and the blood-brain tumor barrier (BBTB), along with superior tumor-targeting performance. We also tracked the GE-C6 NPs in tumor-bearing mice at 1, 6, 24, and 48 h post-injection. The fluorescence signal peaked at 6 h, remained relatively high at 24 h, and then dropped sharply between 24 and 48 h (Fig. S9, Supporting Information), likely due to the degradation of GE-C6 NPs into monomers, which were rapidly metabolized and cleared. In summary, GE NPs showed promising pharmacokinetic properties, maintaining high plasma concentrations for up to 24 h and effectively crossing both the BBB and BBTB to reach the tumor core. Their tumor-targeting efficiency was notably higher than that of PLGA-C6 and free C6, as demonstrated by their superior tumor accumulation. However, improving the tumor retention of GE NPs remains a challenge.

Enhancing tumor-targeting modifications (e.g., angiopep-2 peptide) or extending circulation time (e.g., using red blood cell membrane coating) could potentially increase tumor accumulation and improve therapeutic outcomes.

### 3.3 GE NPs inhibit GBM cell proliferation and promote cell apoptosis

**Fig. 3:**
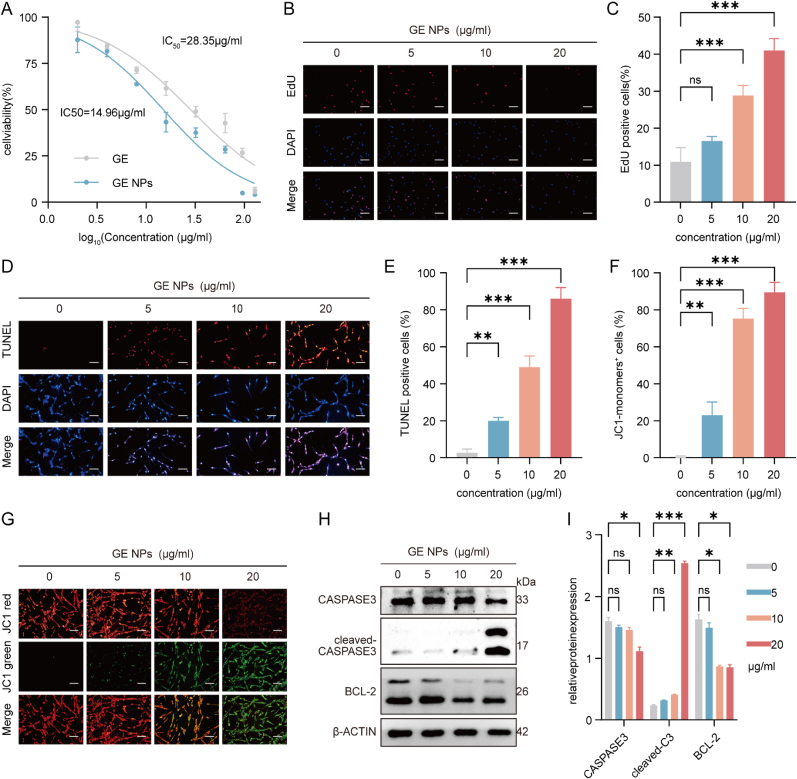
**The role of GE NPs in apoptosis, proliferation in U87MG cells**. (A) Cell viability measured by CCK8 assay after GE and GE NPs treatment with various concentrations in 24h; (B, C) Edu assay showed the DNA synthesis after GE NPs treatment, Scale bars:100 μm; (D, E) TUNEL staining of U87MG after GE NPs treatment, Scale bars:100 μm; (F, G) JC-1 staining solution indicates mitochondrial membrane potential after GE NPs treatment, Scale bars:100 μm; (H, I) Western blot assay validates the expression of apoptosis-related proteins following treatment with GE NPs; Data are presented as mean ± SD. n = 3 per group, ∗p < 0.05, ∗∗p < 0.01, ∗∗∗p < 0.001.

GBM exhibits the key characteristics typical of cancer, such as uncontrolled proliferative signaling, the ability to bypass growth suppressors, resistance to programmed cell death, limitless replicative potential, the promotion of angiogenesis, and the activation of invasion and metastasis [28]. Initial biocompatibility assessment revealed differential cytotoxic profiles between GE and GE NPs in human normal hippocampal neuronal HT22 cells. Quantitative dose-response analysis demonstrated respective half-maximal lethal concentrations (LC_50_) of 97.79 μg/mL for GE and 77.60 μg/mL for GE NPs, indicating a 20.8 % reduction in biosafety threshold following nanoencapsulation (Fig. S10, Supporting Information). To investigate the effects of GE NPs on these characteristics, we first evaluated the impact of GE and GE NPs on the proliferation of U87MG and GL261 cells using the CCK8 assay. The half-maximal inhibitory concentration (IC_50_) values for GE and GE NPs on U87MG cells were found to be 28.35 μg/ml and 14.96 μg/ml, respectively (Fig. 3A), whereas on GL261 cells were 22.54 μg/ml and 18.37 μg/ml (Fig. S11, Supporting Information). We noted that some precipitation occurred when adding GE dissolved in DMSO to the culture medium, which could explain the relatively higher IC_50_ for GE. To further investigate the anti-proliferative effects of GE NPs, we used the EdU assay to assess DNA replication. Low concentrations of GE NPs showed little impact on cell proliferation, but above 10 μg/ml, a dose-dependent inhibition was observed. At 20 μg/ml, GE NPs inhibited nearly 50 % of cell proliferation (Fig. 3B–C).

Through TUNEL staining, we further explored the effect of GE NPs on U87 cell apoptosis. The results found that the proportion of TUNEL positive cells significantly increased after GE NPs treatment (Fig. 3D–E). One early event in apoptosis is the loss of mitochondrial membrane potential. We used JC-1 dye to assess this change, where healthy mitochondria, with high membrane potential, cause JC-1 to form aggregates that emit red fluorescence (JC-1 red). In early apoptosis, as membrane potential declines, JC-1 remains as monomers, emitting green fluorescence (JC-1 green). Our results showed a concentration-dependent decrease in mitochondrial membrane potential with increasing GE NPs doses, indicated by a rise in JC-1 green and a reduction in JC-1 red fluorescence (Fig. 3F–G). Caspase-3, a key executioner in the apoptosis pathway, cleaves intracellular substrates, leading to cytoskeletal breakdown, DNA fragmentation, and apoptotic body formation. Its cleaved form indicates active involvement in apoptosis [29]. Additionally, BCL-2, which helps maintain

mitochondrial membrane integrity and prevent apoptosis [30], was suppressed in a dose-dependent manner by GE NPs, while cleaved caspase-3 levels increased (Fig. 3H–I). These results suggest that GE NPs promote apoptosis by simultaneously activating caspase-3 and downregulating BCL-2 expression.

These findings highlight the potential of GE NPs as a promising therapeutic agent for GBM, targeting multiple cancer hallmarks. However, future studies should focus on optimizing GE NPs for enhanced tumor accumulation and exploring combination therapies to further improve their anti-cancer efficacy.

### 3.4 GE NPs suppress the EMT of GBM

**Fig. 4:**
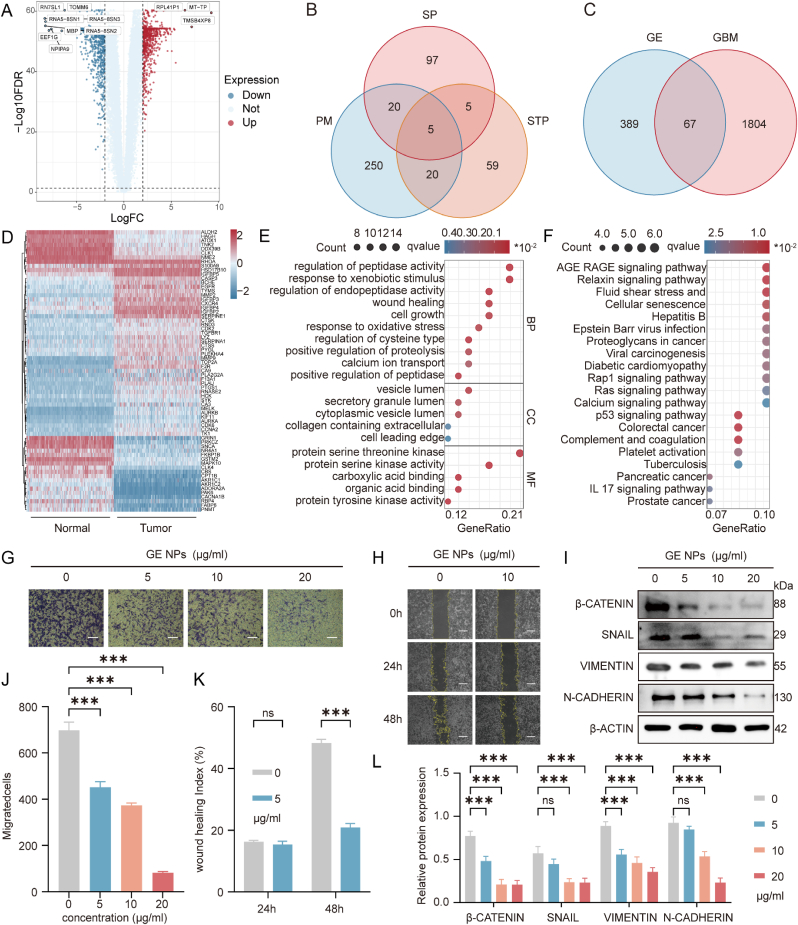
**Investigate the specific phenotypes of GE in combating GBM**. (A)The differentially expressed gene of GBM from TCGA and GTEx databases; (B) The overlap of GE targets in PM, SP and STP databases; (C) The overlap of GE targets and differentially expressed genes of GBM; (D) Heatmap of the 67 GE-related targets expressions; (E) GO analysis of 67 GE-related targets of in GBM; (F) KEGG analysis of 67 GE-related targets in GBM; (G, J) Transwell assay investigates the effect of GE NPs on the invasiveness of U87MG cells, Scale bars:200 μm; (H, K) Scratch assay assesses the migratory capacity of U87MG cells following treatment with GE NPs, Scale bars:400 μm; (I, L) Western blot assay detects the expression of EMT-related proteins following treatment with GE NPs in U87 cells. Data are presented as mean ± SD. n = 3 per group, ∗p < 0.05, ∗∗p < 0.01, ∗∗∗p < 0.001.

In order to further explore the specific phenotype of GE in GBM, we conducted differential expression analysis using RNA sequencing (RNA-seq) data from both GBM and normal tissue samples. The

analysis revealed 1871 genes with significant differential expression in GBM tissues, of which 1044 were downregulated and 827 were upregulated (Fig. 4A, Table S1, Supporting Information, |logFC| > 2). The expression level of the differentially expressed are presented in Table S2 (Supporting Information). Several of the upregulated genes, including EGFR, and VEGFA, are well-established drivers of GBM progression, associated with cell proliferation, invasion, and angiogenesis. The downregulated genes, such as TP53 and CDKN2A, are commonly involved in tumor suppressive pathways. These findings reflect the aggressive nature of GBM and underscore the need to target these key molecular pathways for effective therapy.

To predict the potential targets of GE, we used three drug target databases: STP, SP, and PM, which yielded 99, 137, and 275 associated targets, respectively (Fig. 4B–Table S3, Supporting Information). A Venn diagram was used to illustrate the overlap, and 67 GE-related targets were identified after intersecting these predicted targets with the differentially expressed genes in GBM (Fig. 4C–Table S4, Supporting Information). Their expression levels were then visualized using a heatmap (Fig. 4D). Interestingly, several targets such as MMP9, EGFR, and CDK2 appeared in multiple databases, highlighting their potential relevance in GE's anti-GBM activity.

We performed Gene Ontology (GO) enrichment analysis on the 67 targets to gain insight into the Biological Processes (BP), Cellular Components (CC), and Molecular Functions (MF) that GE might influence (Fig. 4E–Table S5, Supporting Information). In the BP category, the analysis indicated that GE may regulate protease activity, oxidative stress response, cell growth, and calcium ion transport, potentially affecting GBM cell proliferation, migration, invasion, and apoptosis. For the CC category, the analysis suggested that GE might be involved in the regulation of vesicle-mediated secretion and transport, particularly influencing the collagen-containing extracellular matrix. This suggests that GE

may play a role in modifying the tumor microenvironment, potentially reducing GBM cell invasion and migration by altering extracellular matrix (ECM) remodeling and degradation, thereby inhibiting the epithelial-mesenchymal transition (EMT) of GBM cells. EMT is a fundamental process that contributes to cellular plasticity, enhancing the ability of cancer cells to migrate and invade surrounding tissues. In GBM, EMT-like mechanisms have been shown to drive tumor aggressiveness by facilitating cellular detachment, acquisition of stem cell-like properties and remodeling of the ECM [31]. The ECM is composed of various components, including proteoglycans, glycosaminoglycans, structural proteins such as collagen and elastin, adhesion proteins like fibronectin and laminin, and a group of enzymes known as matrix metalloproteinases (MMPs) [32]. In the MF analysis, GE appeared to mainly interfere with the activity of enzymes such as serine, threonine, and tyrosine kinases, which are linked to key signaling pathways like MAPK/ERK and PI3K/AKT/mTOR. These pathways are known to promote cell survival and growth in GBM, suggesting that GE's ability to inhibit these kinases could reduce tumor progression. Additionally, KEGG pathway enrichment analysis revealed that the 67 targets were enriched in cancer-related pathways, such as the Relaxin signaling pathway and proteoglycan networks in cancer, both of which are involved in extracellular matrix remodeling. Other significant pathways, including Rap1, Ras, and p53 signaling, implicated GE in the regulation of cell proliferation and apoptosis (Fig. 4F–Table S6, Supporting Information).

The EMT of GBM is involved shift in the balance of protein networks like E-cadherin, N-cadherin, Vimentin, cell polarity complexes, and proteases [33]. To examine the effects of GE NPs on EMT, we first assessed cell invasion using a transwell assay, which showed a dose-dependent reduction in the number of invading cells with increasing GE NPs concentrations (Fig. 4G and J). We further used a wound healing assay to measure U87MG cell migration. GE NPs significantly reduced wound closure after 48 h (Fig. 4H and K). Western blot analysis for EMT-related proteins (β-catenin, SNAIL, Vimentin, and N-cadherin) indicated that their expression decreased w ith higher GE NPs doses (Fig. 4I and L, Figure.S12, Supporting Information). The reduction in Vimentin and other EMT markers suggests that GE NPs inhibit EMT, thereby limiting GBM cell migration and invasion.

### 3.5 GE NPs suppress the EMT of GBM by targeting the catalytic domain of MMP9

**Fig. 5:**
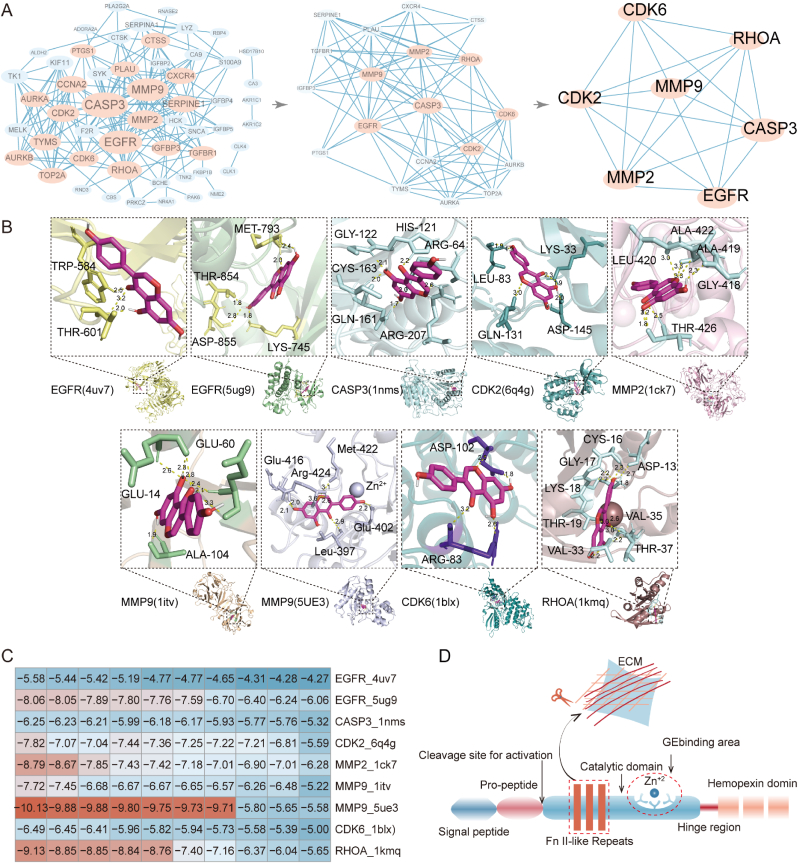
**Exploring the Targets of GE in GBM**. (A) PPI network analysis of GE-relative targets in GBM; (B) Molecular docking results of seven hub targets with GE; (C)Heatmap of binding energy for GE to seven hub targets; (D) Schematic illustration of MMP9 protein structure and the binding site of MMP9.

To deepen our understanding of GE's molecular mechanisms in GBM, we conducted a PPI network analysis of the 67 GE-related targets using the STRING database (Fig. S13, Table S7, Supporting Information). This analysis identified 54 core genes, which were further analyzed for node centrality using the cytoNCA plugin in Cytoscape, resulting in the selection of seven hub genes: RHOA, CASP3, MMP2, MMP9, CDK2, CDK6, and EGFR (Fig. 5A, Fig. S14, Table S8, Supporting Information). Each of these hub genes plays a distinct role in GBM biology:

**RHOA**: Involved in cytoskeletal organization and cell motility, contributing to tumor invasion [34];

**CASP3**: Acts as an executioner in apoptosis, promoting programmed cell death in cancer cells [29];

**MMP2 and MMP9**: Play a crucial role in the degradation of the extracellular matrix, facilitating tumor

cell invasion. Specifically, MMP9, a zinc-dependent protease, breaks down components of the extracellular matrix, disrupting cell adhesion and epithelial structure, thereby promoting EMT [35,36];

**CDK2 and CDK6**: Regulate cell cycle progression, influencing GBM cell proliferation [37];

**EGFR**: A well-known driver of cell growth and survival, often overexpressed in GBM [38];

These hub genes indicate potential therapeutic targets for GE, as modulating their activity could interfere with critical pathways driving GBM progression.

We performed molecular docking of GE with the seven core target proteins using AutoDock software to evaluate binding affinity (Fig. 5B). Among the docking results, GE showed the most stable binding of MMP9 (5ue3), with a binding energy of −10.13 kcal/mol (Fig. 5C). This binding involved the formation of hydrogen bonds with residues Leu^397^, Glu^402^, Glu^416^, Met^422^, and Arg^424^(Fig. S15, Supporting Information). MMP9, a member of the gelatinase B family, is among the most intricate matrix metalloproteinases. It is predominantly expressed in the hippocampus, cerebellum, and cerebral cortex, and is secreted by a range of cell types, including endothelial cells, leukocytes, fibroblasts, neutrophils, and macrophages [39]. The MMP9 gene is located on chromosome 20q13.12 and is composed of 13 exons and 12 introns. Structurally, MMP9 includes several essential domains: a signal peptide, a propeptide region, a catalytic domain, a hemopexin-like domain, and a hinge region (Fig. 5D). The catalytic domain, spherical and comprising 170 amino acids, facilitates the enzyme's proteolytic activity [40]. It contains a critical zinc-binding motif (HEXXHXXGXXH), where Glu^402^ plays a pivotal role in enzymatic function [41]. Two zinc ions within this domain ensure catalytic efficiency and structural stability, while five calcium ions enhance the overall stability of the enzyme. The catalytic domain further divides into N-terminal and C-terminal regions, separated by a shallow catalytic groove and linked by a U-shaped loop [42]. This groove harbors six binding pockets: three on the left side (S1, S2, and S3) and three on the right (S1', S2', and S3'), encircling the catalytic zinc ion [43]. The substrate specificity of MMP9 is largely determined by the dimensions, depth, and amino acid composition of the S1 pocket, which varies across different MMP family members [44]. MMP9 features an intermediate-sized S1 pocket, similar in position, size, and solvent exposure to that of MMP2, with a key difference being a loop structure formed by residues 425–431 in MMP9, which is absent in MMP2 [40]. A unique characteristic of MMP9 and MMP2 is the presence of a fibronectin domain, composed of three fibronectin type II motifs inserted into the metalloproteinase domain [45]. This domain is essential for recognizing, binding, and degrading substrates such as gelatin, laminin, and collagen types I and IV. Structural studies of MMP9 co-crystallized with various MMP inhibitors (MMPi) have shown that the Arg424 residue is highly flexible, sometimes moving to a position that blocks the S1 pocket. This occlusion explains why certain. MMPi with long P1' substituents are less effective at inhibiting MMP9 compared to MMP2 [46]. In summary, these findings suggest that GE may inhibit the degradation of the extracellular matrix by interacting with the catalytic domain of MMP9, thereby suppressing the EMT process in GBM cells.

**Fig. 6:**
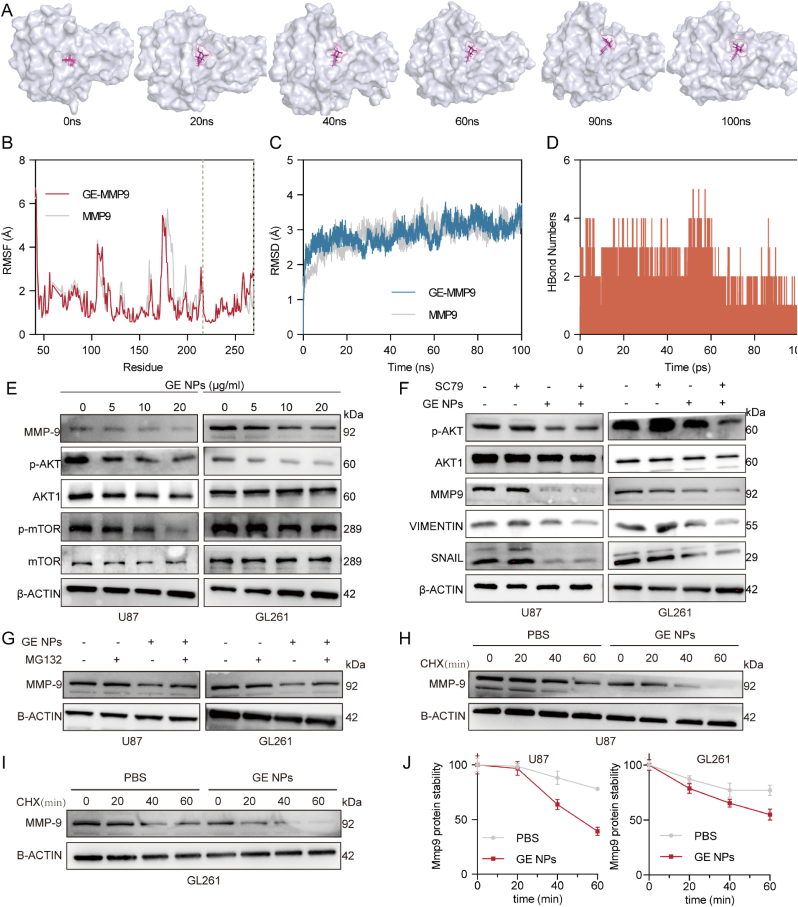
**Validate the stability of the interaction between GE and MMP9**. (A) The binding patterns between the small molecule and the protein (GE-MMP9) at different time points during the MD simulation; (B) RMSF calculated based on MD simulation trajectories; (C) Time-dependent changes in the RMSD of the MMP9 and GE-MMP9 complex during MD simulation; (D) Changes in the number of hydrogen bonds between GE and MMP9 during MD simulation; (E) Western blot analysis investigating the effects of GE NPs on the protein levels of MMP9 and the AKT/mTOR signaling pathway in U87MG and GL261 cells; (F) Western blot analysis of the phosphorylation levels of AKT, MMP9 and the EMT-related proteins following treatment with GE NPs and SC79; (G U87 and GL261 cells were treated with GE NPs (0 or 10 μg/mL), followed by MG132 treatment, and MMP9 protein levels were assessed; (H, I, J) GL261 cells were treated with OA NPs (0 or 30 μg/mL), followed by CHX treatment, and protein extracts were collected for Western blotting at the designated time points. n = 3 per group, Data are presented as mean ± SD.

To further assess the stability of GE binding to MMP9, molecular dynamics simulations were conducted. Specifically, to verify the stability of the small molecule within the active site, we extracted complex conformations at 0, 20, 40, 60, 80, and 100 ns (Fig. 6A). Throughout the simulation, GE remained stably anchored in the binding pocket, indicating a sustained interaction with MMP9. Initially, GE fit snugly into the pocket, but as the simulation progressed, the pocket gradually underwent conformational adjustments, reaching equilibrium around 60 ns, while GE remained tightly bound. This suggests that the GE-MMP9 interaction remains stable, particularly during the mid-to-late stages of the simulation. In addition, Root Mean Square Fluctuation (RMSF) analysis, which reflects protein flexibility, revealed that lower RMSF values indicated higher stability in specific regions. Following the binding of GE, MMP9 exhibited decreased RMSF values, suggesting that the interaction stabilized the protein. Notably, the RMSF fluctuations at the GE binding site, particularly near the zinc-binding region (between the dashed lines, Fig. 6B), were minimal. Furthermore, the overall RMSF values of the GE-MMP9 complex closely aligned with those of MMP9 alone, indicating the formation of a stable complex. Similarly, Root Mean Square Deviation (RMSD) analysis, which measures the overall motion of the complex, showed no significant fluctuations in the GE-MMP9 complex compared to MMP9 alone (Fig. 6C). This lack of significant RMSD variation suggests that the protein's structure remained intact, providing a stable foundation for GE binding. Moreover, the radius of gyration (Rg), which reflects the compactness of the system, exhibited fluctuations in the GE-MMP9 complex similar to those of MMP9, stabilizing around 17.7 Å (Fig. S16, Supporting Information). However, a notable increase in Rg occurred around 60 ns, followed by a gradual decrease, potentially indicating a

transient change at the binding site. Despite this fluctuation, the complex maintained a compact structure. The solvent-accessible surface area (SASA), which measures the surface area of the complex exposed to solvent, also remained generally stable throughout the simulation. However, the SASA of the GE-MMP9 complex was slightly higher than that of MMP9 after 40 ns, suggesting a minor conformational change due to GE binding (Fig. S17, Supporting Information). By 100 ns, the SASA returned to levels similar to MMP9, indicating no significant structural changes in the final conformation. Hydrogen bonding, a critical non-covalent interaction, was monitored throughout the 100 ns simulation. The GE-MMP9 complex exhibited between 0 and 5 hydrogen bonds, typically around 2, highlighting the role of hydrogen bonding in maintaining the stable binding of GE to MMP9 (Fig. 6D). Finally, the binding free energy between GE and MMP9 was calculated using the. MM/PBSA method, yielding a value of −29.048 kcal/mol. This negative value indicates strong binding affinity, with lower free energy correlating to stronger binding. Thus, the results confirm that GE has a high affinity for MMP9. In summary, the molecular dynamics simulations indicate that GE forms a stable complex with MMP9, as evidenced by consistent hydrogen bonding, minimal RMSD fluctuations, and favorable binding free energy. These findings suggest that GE has a strong and stable binding affinity to MMP9, supporting its potential as an effective inhibitor.

To validate the molecular targets, we performed western blot analysis to measure the expression of MMP9 and components of the AKT/mTOR pathway in U87 and GL261 cells treated with GE NPs. The results showed a dose-dependent decrease in the expression of p-AKT, p-mTOR, and MMP9 (Fig. 6E, Fig. S18, Supporting Information), indicating that GE NPs could inhibit the activation of the AKT/mTOR pathway in GBM cells. To further investigate whether the reduction in MMP9 expression was due to inhibition of the AKT/mTOR pathway, we conducted rescue experiments using the AKT agonist SC79. SC79 reversed the GE NPs-induced decrease in p-AKT levels but did not restore the expression of MMP9, Vimentin, or SNAIL (Fig. 6F, Fig. S19, Supporting Information). Analysis of MMP9 protein levels following CHX and MG132 treatments showed that when protein degradation was blocked (MG132 treatment), MMP9 levels remained unchanged after GE NPs treatment (Fig. 6G, Fig. S20, Supporting Information). However, when transcription was inhibited (CHX treatment), GE NPs accelerated the reduction of MMP9 protein levels (Fig. 6H–J). This suggests that the inhibitory effect of GE NPs on MMP9 may be achieved by promoting its rapid degradation. These findings indicate that GE NPs regulate MMP9 levels independently of the AKT/mTOR pathway, suggesting a distinct mechanism by which GE NPs influence GBM cell EMT process.

### 3.6 In vivo antitumor activity of GE NPs

**Fig. 7:**
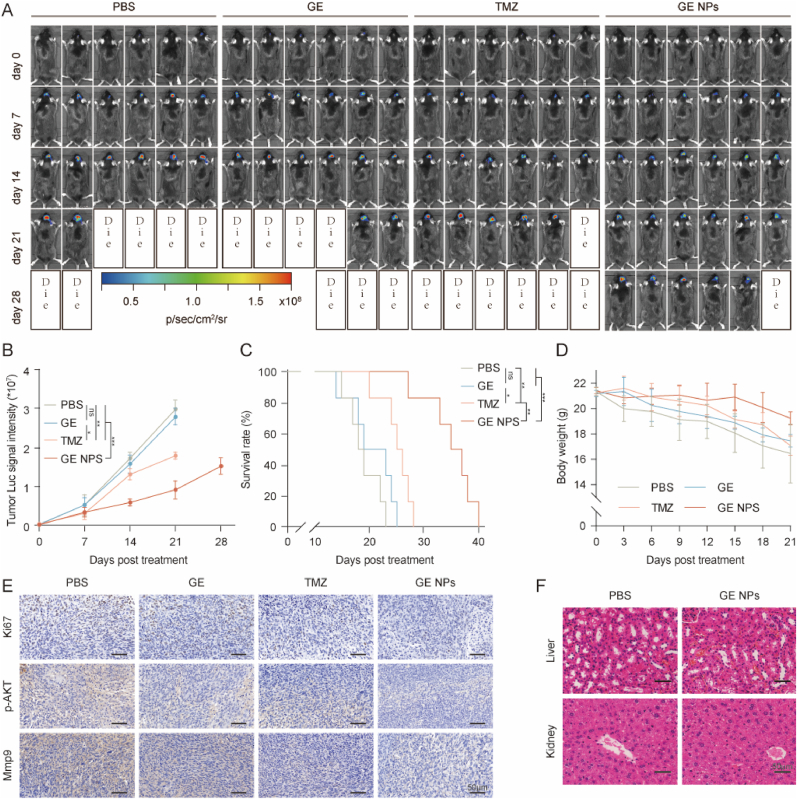
**In vivo antitumor efficacy of GE NPs in C57BL/6J mice**. (A) IVIS bioluminescence imaging of orthotopic GL261-Luc GBM mice from each group during treatment; (B) Mean GL261-Luc tumor luminescent signal intensity of mice in different treatment groups; (C) Kaplan–Meier survival of mice in different treatment groups; (D) Body weight changes of mice (E) GL261-Luc tumors from different treatment groups were immunostained with Ki67, MMP9 and p-AKT; (F) H&E staining of major organs harvested from GL261-Luc bearing mice. n = 6 per group, Data are presented as mean ± SD, (∗p < 0.05, ∗∗p < 0.01, ∗∗∗p < 0.001).

The in vivo antitumor efficacy of GE NPs was evaluated in C57BL/6J mice bearing GL261-Luc orthotopic xenograft tumors. Mice were treated with GE (20 mg/kg), delivered via intravenous injection every three days (n = 6, Fig. S21, Supporting Information). Treatment groups included GE, temozolomide (TMZ), GE NPs, and PBS (control). Tumor volume was monitored by measuring bioluminescence emitted from GL261-Luc cells, using the bioluminescence intensity on day 0 as a reference. The results showed that both PBS and GE failed to suppress tumor growth (Fig. 7A–B). In contrast, mice treated with GE NPs exhibited a significant reduction in tumor growth compared to the PBS, GE, and TMZ groups, indicating that GE NPs possess superior BBB and blood-brain tumor barrier (BBTB) penetration capabilities, along with deep tumor penetration, which effectively inhibited tumor growth.

The overall survival across the different treatment groups was also assessed. As expected, mice treated with GE NPs had the longest median survival time among all groups (Fig. 7C). The median survival times were as follows: GE NPs (36 days) > TMZ (25.5 days) > GE (21 days) > PBS (18.5 days) (Table S9, Supporting Information). Furthermore, mice in the GE NPs group maintained a relatively stable body weight, with only minor weight loss observed during the treatment period, compared to the PBS and GE groups (Fig. 7D). To further evaluate the effects of GE NPs on tumor biology, immunohistochemical (IHC) staining was performed on GBM tissue sections for proliferation marker Ki67, MMP9, and p-AKT). The results showed that GE NPs treatment significantly reduced the expression of Ki67, MMP9, and p-AKT compared to the PBS, GE, and TMZ groups, suggesting that GE NPs effectively suppressed tumor cell proliferation and extracellular matrix degradation (Fig. 7E). Importantly, no significant histological damage to major organs was observed in mice treated with GE NPs (Fig. 7F, Fig. S22, Supporting Information), indicating that GE NPs exhibited good biocompatibility and safety. Moreover, we further assessed the biocompatibility and systemic response of the GE NPs by measuring blood biochemical indexes at 24 h after one single injection, including plasma alanine aminotransferase (ALT), aspartate aminotransferase (AST), plasma urea (BUN), creatinine (Cre) and routine blood test. Mice treated with GE NPs had relatively stable biochemical parameters (Fig. S23, Table S10, Supporting Information)

## 4 Conclusion

This study demonstrates that GE NPs effectively address the limitations of free GE, such as poor bioavailability and limited tumor targeting, by enhancing stability, cellular uptake, and BBB penetration. GE NPs showed significant in vitro and in vivo anti-GBM effects, inhibiting cell proliferation, epithelial-mesenchymal transition (EMT), and promoting apoptosis. They exhibited superior tumor accumulation and prolonged circulation compared to free GE and PLGA, leading to reduced tumor growth and improved survival in GBM-bearing mice. Mechanistically, GE NPs targeted the catalytic domain of MMP9, with molecular simulation revealing strong interactions involving the formation of hydrogen bonds with Glu^402^ and Arg^424^, suggesting inhibition of extracellular matrix degradation. In vivo results showed significant reductions in tumor proliferation markers (Ki67), highlighting their therapeutic efficacy. GE NPs also demonstrated good biocompatibility, with minimal toxicity to major organs. Overall, GE NPs offer a promising therapeutic approach for GBM by addressing critical barriers in drug delivery and improving antitumor efficacy. Future studies should focus on optimizing tumor retention and exploring combination therapies for enhanced treatment outcomes.

## CRediT authorship contribution statement

**Qingyu Zhao:** Writing – original draft, Methodology, Investigation, Formal analysis, Data curation, Conceptualization. **Yong Li:** Validation, Methodology, Formal analysis. **Qian Sun:** Visualization, Validation, Investigation. **Ronggui Wang:** Formal analysis. **Haoran Lu:** Investigation. **Xinyi Zhang:** Investigation. **Lun Gao:** Conceptualization. **Qiang Cai:** Conceptualization. **Baohui Liu:** Writing – review & editing, Supervision, Resources, Conceptualization. **Gang Deng:** Writing – review & editing, Resources, Funding acquisition, Conceptualization.

## Ethics approval and consent to participate

All animal protocols were approved by the Institutional Animal Care and Use Committee of Renmin Hospital of Wuhan University (Ethical Permit Number: WDRM20231002A).

## Funding

This work was supported by the 10.13039/501100001809National Natural Science Foundation of China (82001311) and the Knowledge Innovation Program of Wuhan-Shuguang Project (No. 2022020801020483).

## Declaration of competing interest

The authors declare that they have no known competing financial interests or personal relationships that could have appeared to influence the work reported in this paper.

## Data Availability

Data will be made available on request.
