# Supplementary material for: Self-assembled genistein nanoparticles suppress the epithelial-mesenchymal transition in glioblastoma by targeting MMP9
Source: Mater Today Bio. 2025 Feb 27;31:101606. doi: 10.1016/j.mtbio.2025.101606 (PMC11919400; doi:10.1016/j.mtbio.2025.101606)
Supplement: Multimedia component 1 [file mmc1.pdf]

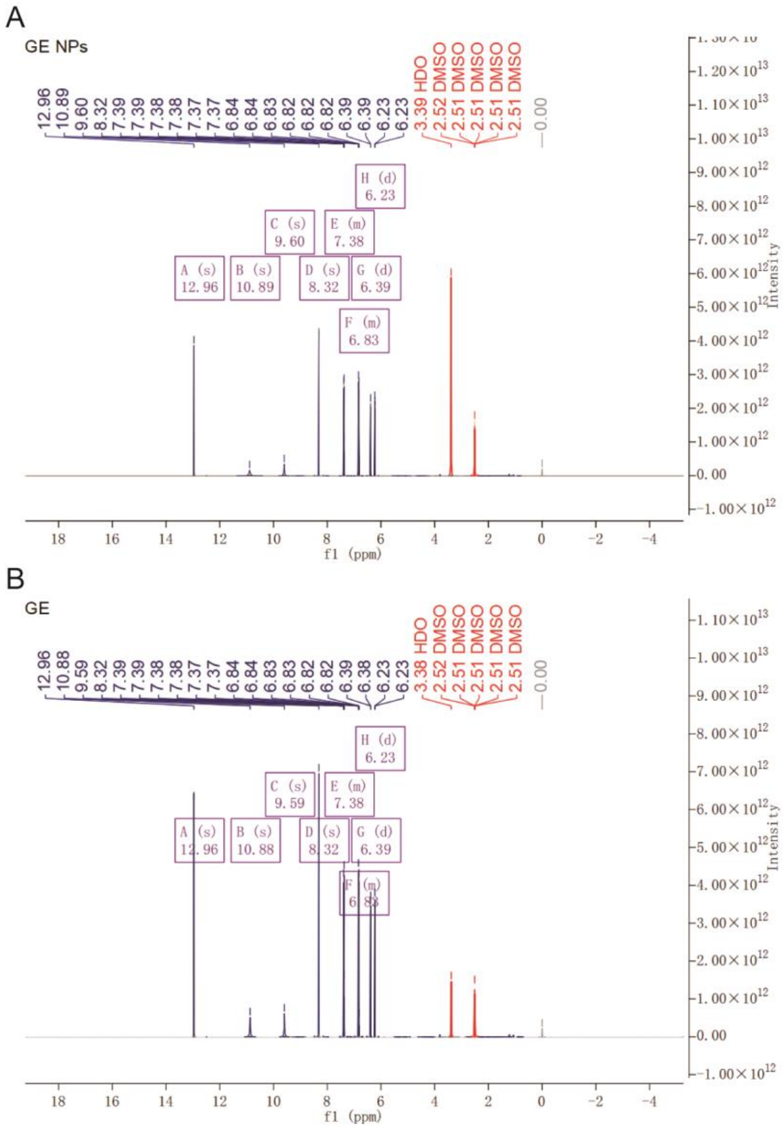

Figure.S1 1H NMR spectrum of GE and GE NPs

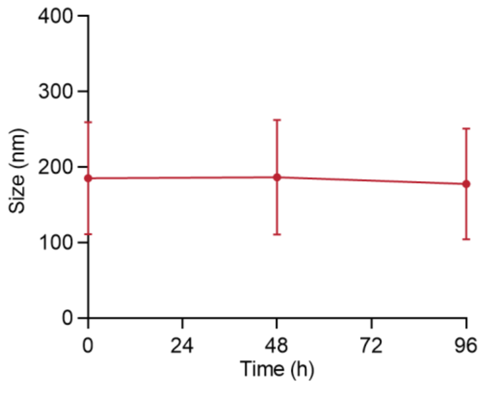

Figure.S2 Long-term structure stability of the GE NPs

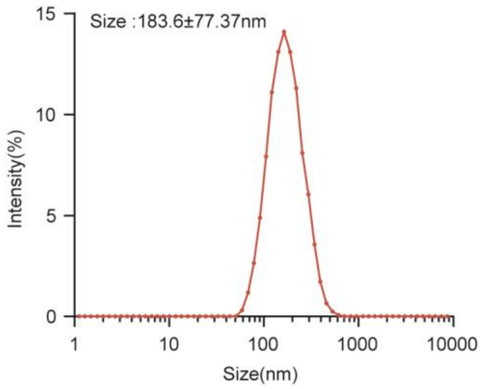

Figure.S3 Mean size of GE NPs in FBS after 24 hours

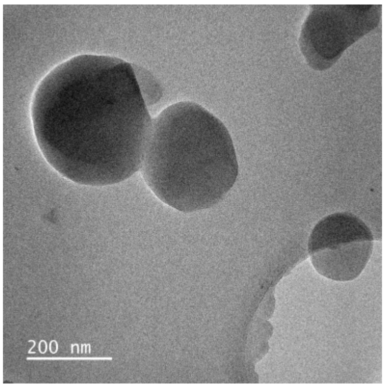

Figure.S4 TEM image of GE-C6 NPs

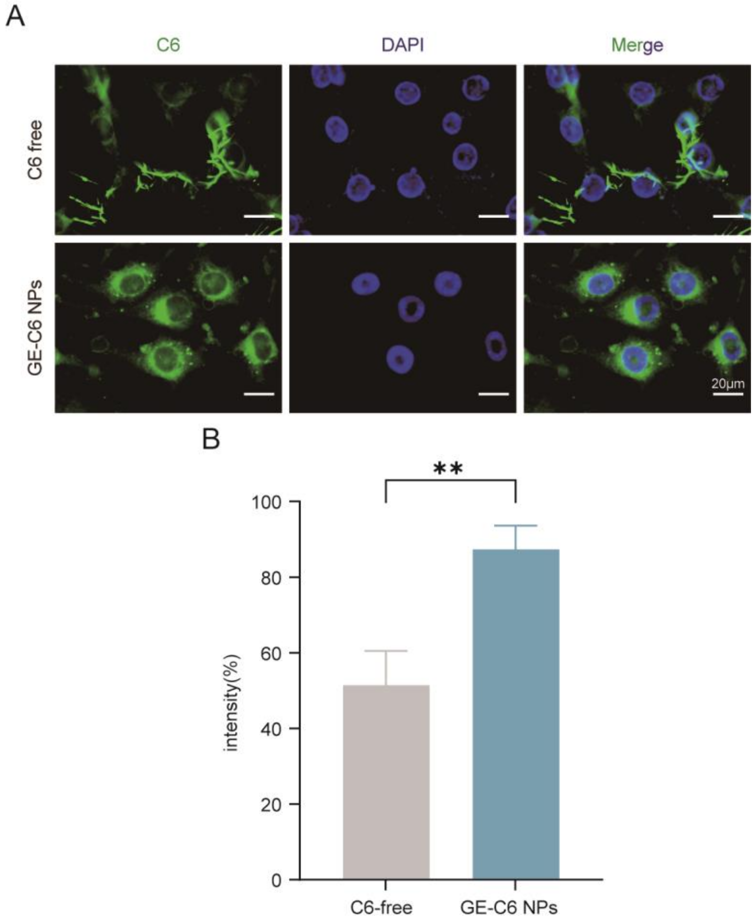

Figure.S5 (A, B) Fluorescence image and intensity of C6 free and GE-C6 NPs in GL261 cells. Scale bar = 20μm, n = 3, **P<0.01.

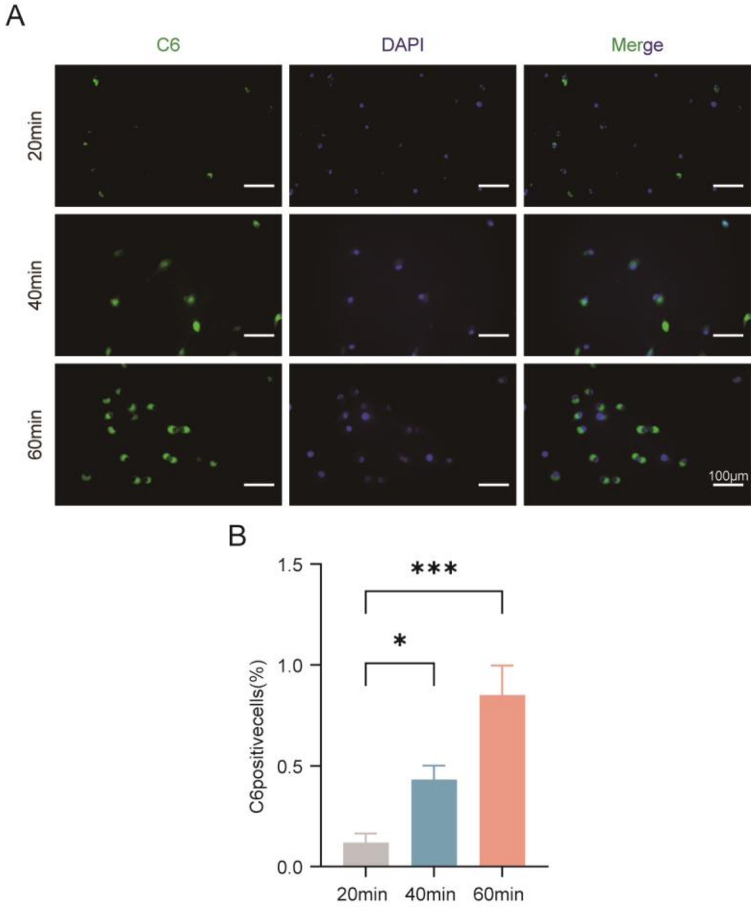

Figure.S6 (A, B) Fluorescence image of GE-C6 NPs in GL261 cells over different time. Scale bar = 100μm. n = 3, *P<0.05, ***P<0.001.

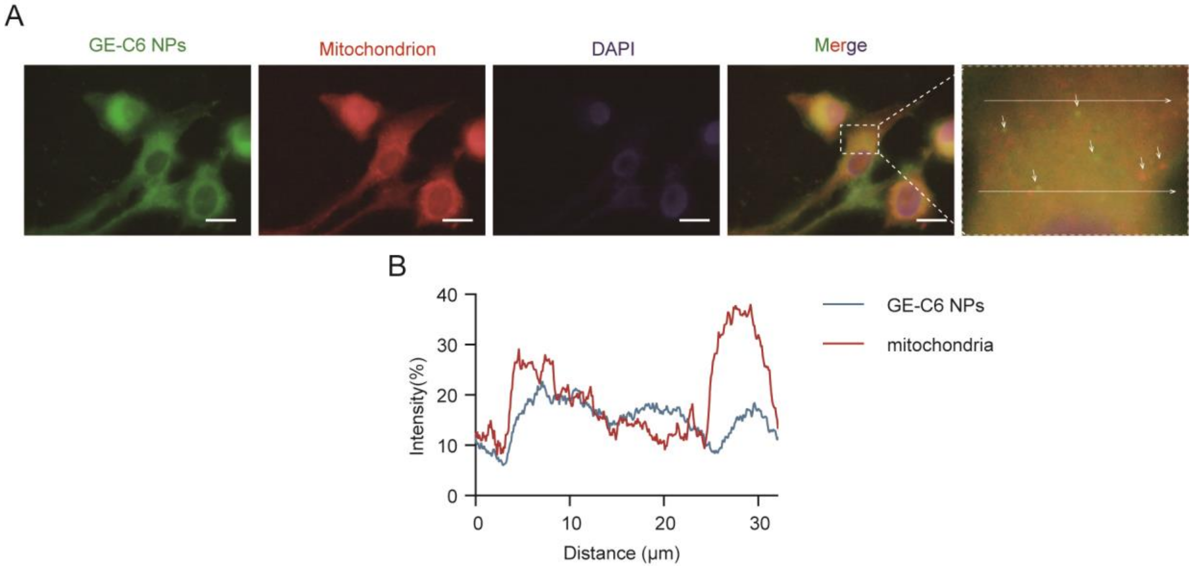

Figure.S7 (A, B) Fluorescence image and intensity of GE-C6 NPs or mitochondria co-localization. Scale bar = 10μm.

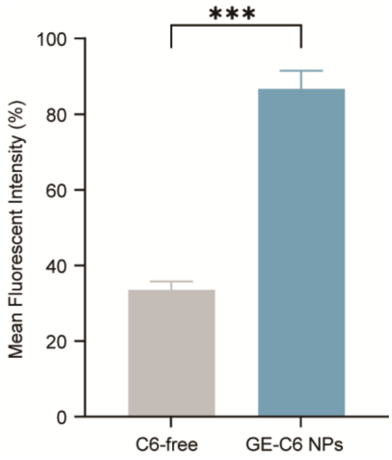

Figure.S8 Fluorescence intensity of C6 free or GE-C6 NPs in GL261 cells. n = 3, ***p<0.001.

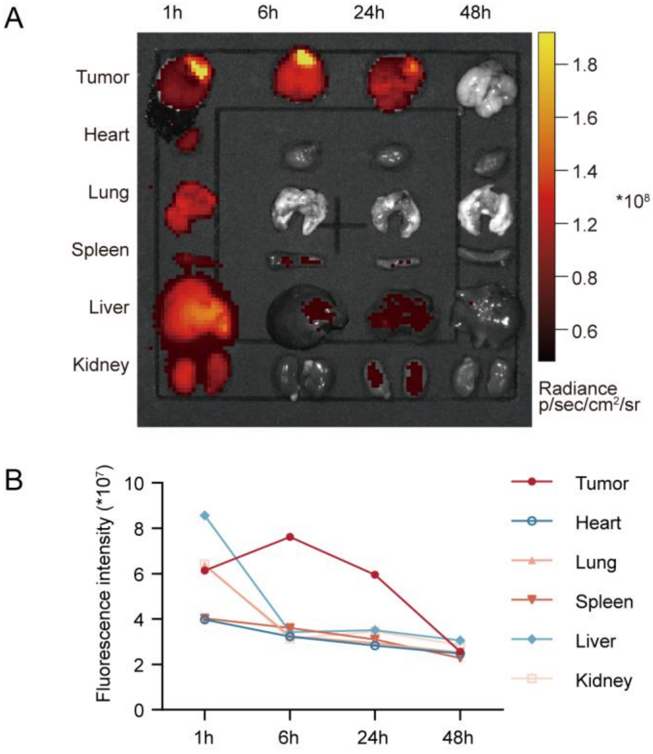

Figure.S9 (A) Ex vivo GE-C6 NPs fluorescence imaging of the harvested organs by an IVIS system at 1, 6, 24 ,48 hours post-injection. Scale bar = 10μm. (B) Fluorescence intensity of harvested organ by IVIS system. n = 3.

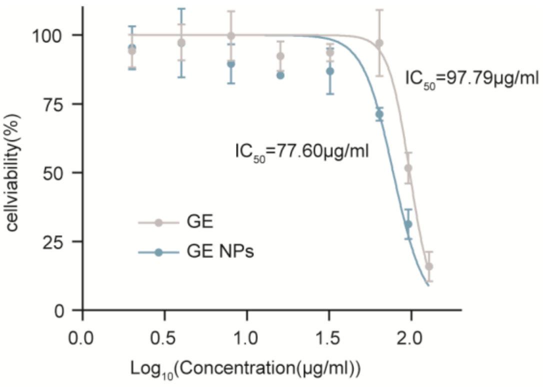

Figure.S10 Cell viability measured by CCK8 assay after GE and GE NPs treatment on HT22 cells.

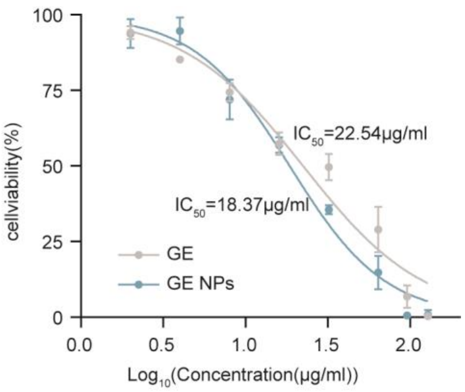

Figure.S11 Cell viability measured by CCK8 assay after GE and GE NPs treatment on GL261 cells.

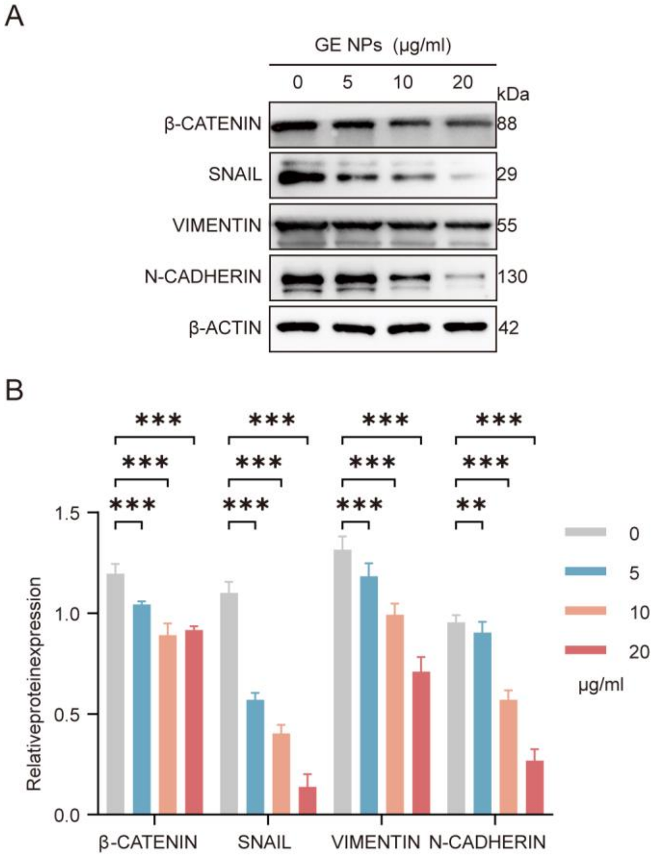

Figure.S12 (A, B) Western blot assay detects the expression of EMT-related proteins following treatment with GE NPs in GL261 cells. n = 3 per group, Data are presented as mean± SD, **p < 0.01, ***p < 0.001.

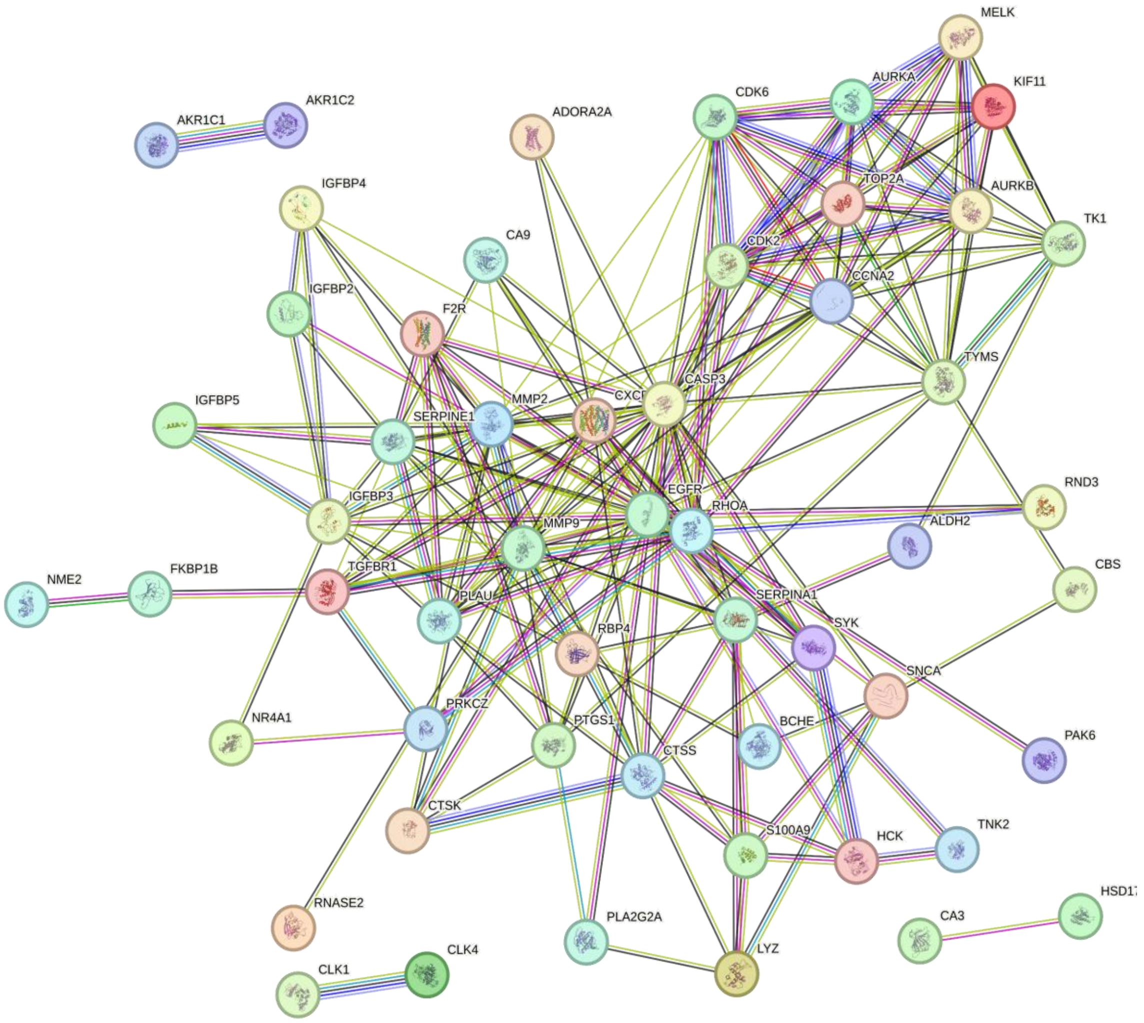

Figure.S13 The PPI network of GE-relative targets

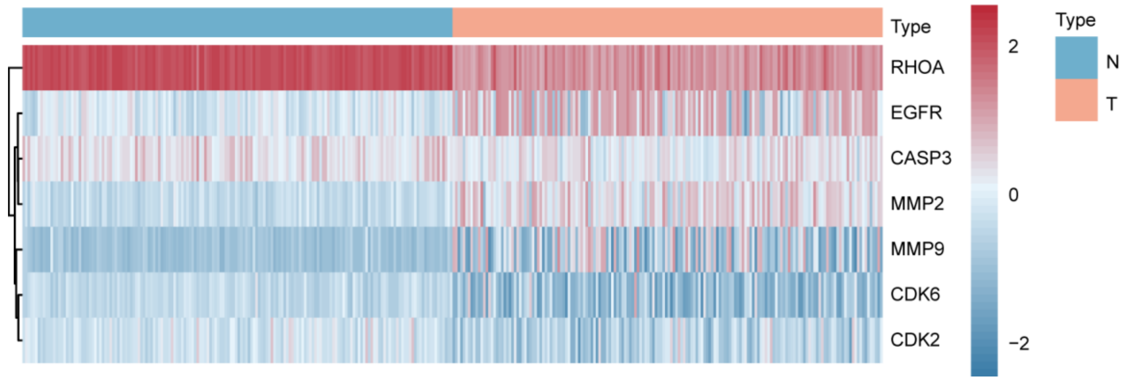

Figure.S14 The heatmap of seven hub genes.

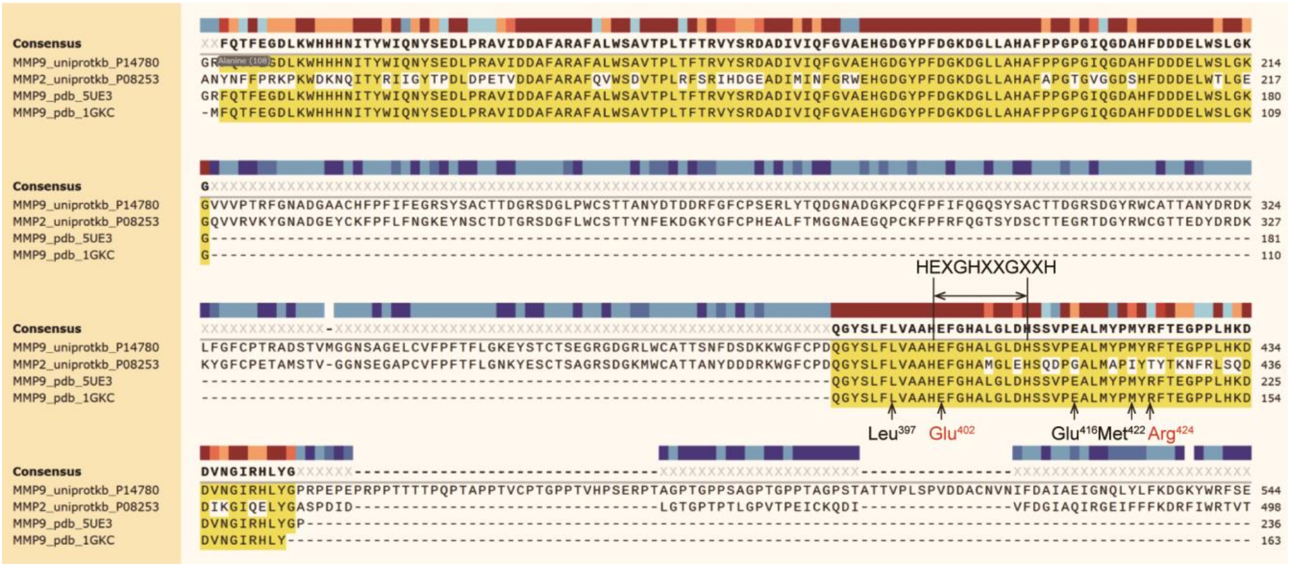

Figure.S15 The sequence alignment results of MMP9, MMP2, and the protein 5ue3 from PDB.

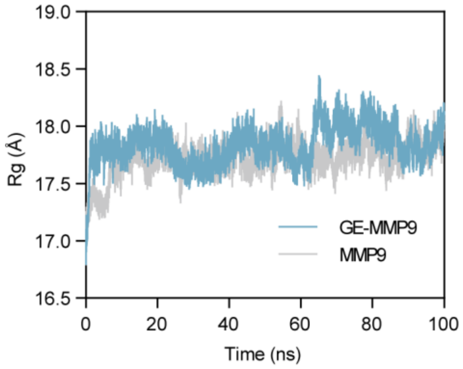

Figure.S16 Changes in the Rg of the MMP9 and GE-MMP9 complex during MD simulation

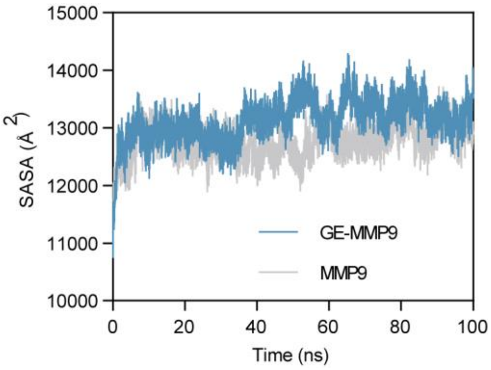

Figure.S17 SASA of MMP9 and GE-MMP9 complex during MD simulation;

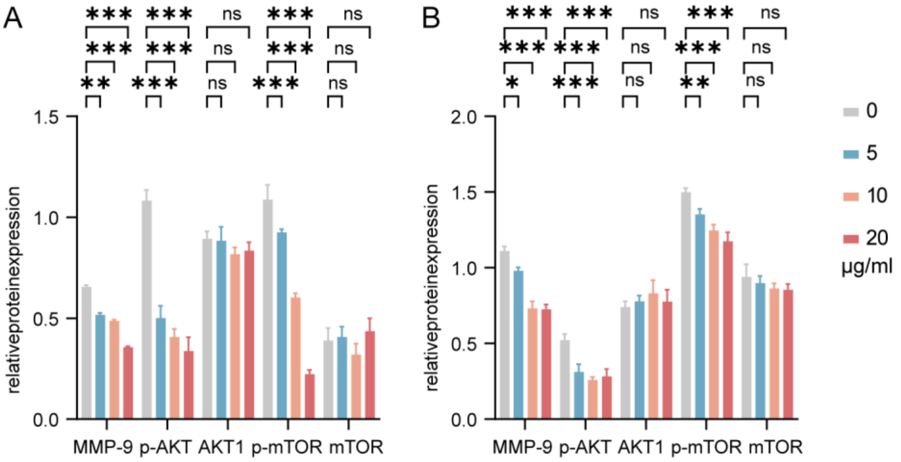

Figure.S18 Western blot analysis investigating the effects of GE NPs on the protein levels of MMP9 and AKT/mTOR pathway. (A) U87 cells; (B)GL261 cells. n = 3 per group, Data are presented as mean± SD, *p < 0.05, **p < 0.01, ***p < 0.001.

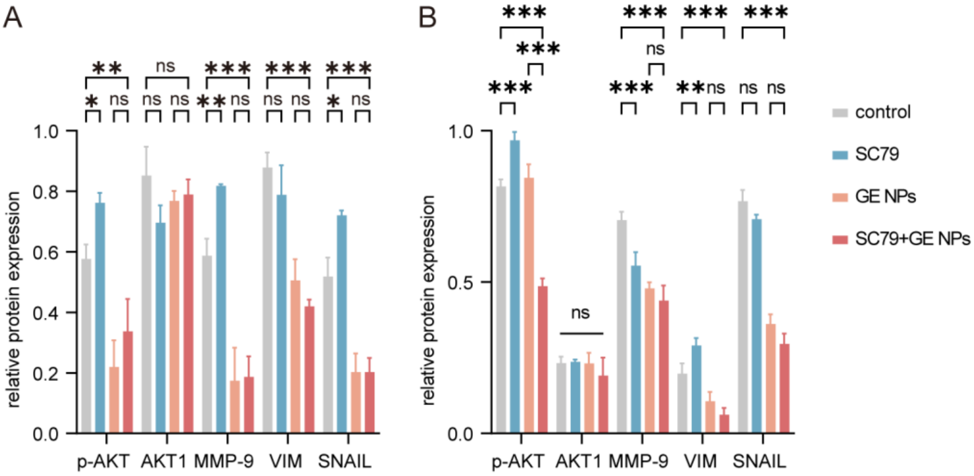

Figure.S19 Western blot analysis of the phosphorylation levels of AKT, MMP9 and the EMT-related proteins following treatment with GE NPs and SC79. (A) U87 cells; (B)GL261 cells. n = 3 per group, Data are presented as mean± SD, *p < 0.05, **p < 0.01, ***p < 0.001.

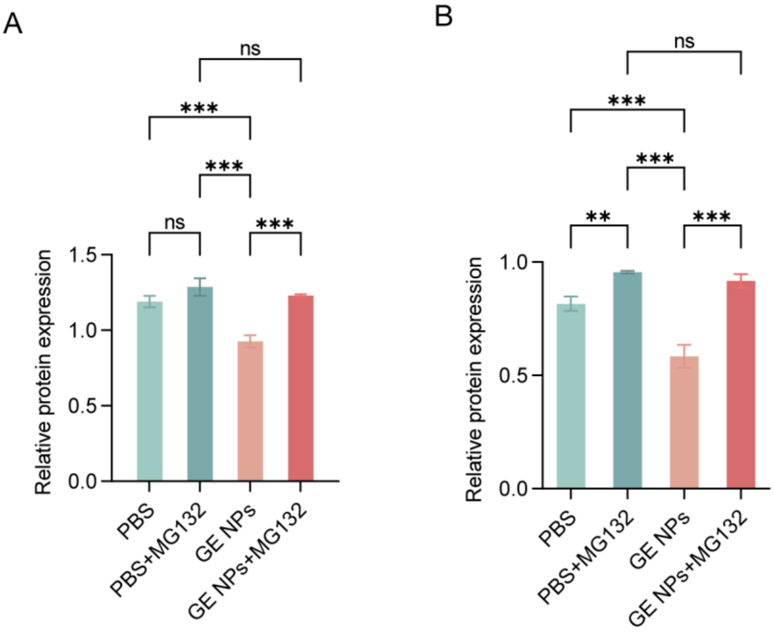

Figure.S20 U87 (A) and GL261 (A) cells were treated with GE NPs (0 or 10 μg/mL), followed by MG132 treatment, and MMP9 protein

levels were assessed. n = 3 per group, Data are presented as mean± SD, **p < 0.01, ***p < 0.001.

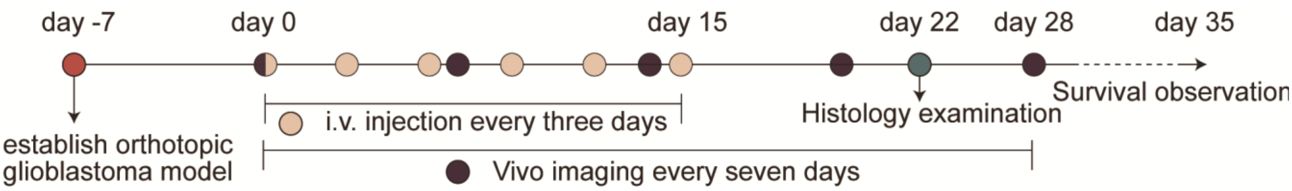

Figure.S21 Schematic illustration showing the timeline of the treatment schedule.

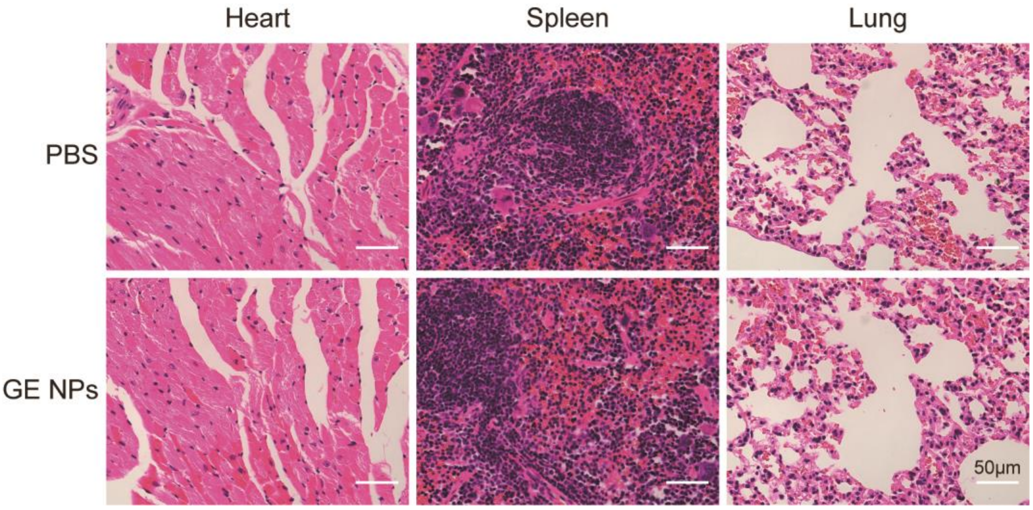

Figure.S22 H&E staining of major organs harvested from GL261-Luc bearing mice.

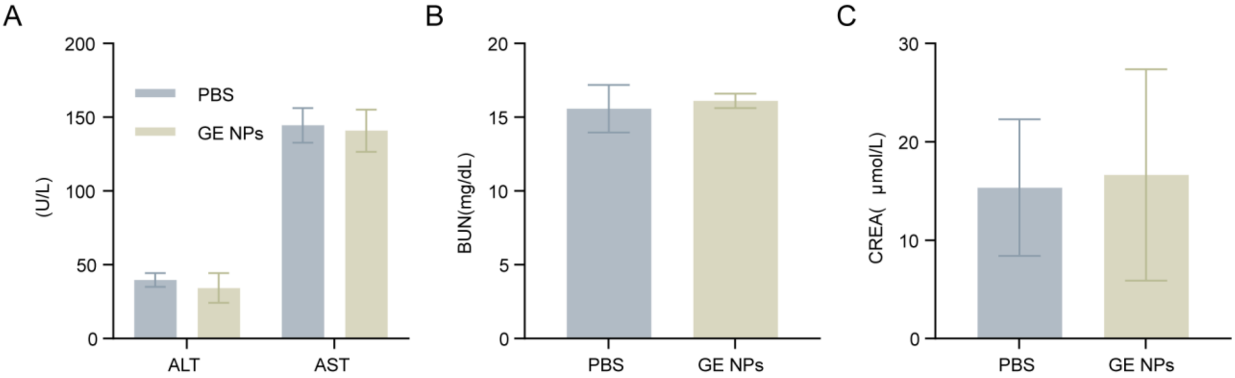

Figure.S23 The effect of GE NPs on the cytotoxicity of the liver and kidney. (A) AST and ALT; (B) BUN and (C) CREA in serum of the healthy C57BJ/6L mice intravenously injected to PBS and GE NPs. Data are presented as mean±SD and n=3.
